# The Association of Chemokine Gene Polymorphisms with VKH and Behcet's Disease in a Chinese Han Population

**DOI:** 10.1155/2017/1274960

**Published:** 2017-05-14

**Authors:** Yang Huang, Hongsong Yu, Qingfeng Cao, Jing Deng, Xinyue Huang, Aize Kijlstra, Peizeng Yang

**Affiliations:** ^1^The First Affiliated Hospital of Chongqing Medical University, Chongqing Key Laboratory of Ophthalmology and Chongqing Eye Institute, Chongqing, China; ^2^University Eye Clinic Maastricht, Maastricht, Netherlands

## Abstract

To investigate the association of chemokine gene polymorphisms and Behcet's disease (BD) and Vogt Koyanagi Harada (VKH) disease in a Chinese Han population. A case-control study was performed. Three hundred and seventy-one BD patients, 371 VKH disease patients, and 605 healthy controls were recruited to determine genetic variants of 26 SNPs in 12 chemokine genes with iPLEX Gold genotyping assay and Sequenom MassARRAY or TaqMan SNP assays. In this study, *P*_uncorr_ values showed a weak association of five SNPs of five genes in BD and three SNPs of three genes in VKH disease. However, after Bonferroni correction, the 26 investigated SNPs showed no significant differences in genetic variants, including genotype and allele frequencies, between BD or VKH disease patients and healthy individuals. Haplotype analysis for the chemokine genes showed a significant association with the TC haplotype of* CXCL12* in VKH. Stratified gender analysis and genotype-phenotype analysis were conducted to analyze the association of the 26 SNPs of 12 chemokine genes with BD and VKH disease. However, no significant association was observed after Bonferroni correction. This study showed no association of 26 SNPs in 12 chemokine genes with both BD and VKH disease in a Chinese Han population.

## 1. Introduction

Uveitis is an intraocular inflammatory disease causing severe visual impairment worldwide [1]. In China, Behcet's disease (BD) and Vogt-Koyanagi Harada (VKH) disease have the highest incidence in uveitis entities. BD is a chronic, relapsing, multisystemic inflammatory disorder, and its classical clinical characters include oral aphthae, genital ulcers, and recurrent iridocyclitis with hypopyon, which is probably due to an autoimmune response [2]. VKH disease is a multisystem autoimmune disease with a hallmark of diffuse granulomatous uveitis accompanied with poliosis, vitiligo, alopecia, and central nervous system abnormalities [3]. Various genes have been demonstrated to be relevant to different types of uveitis, comprising* HLA-B27*,* HLA-A29*,* HLA-B51*,* HLA-DR4*,* IL-10*,* STAT4*,* STAT3, *and* UBAC2* [4–6] which suggested genetic factors are involved in the occurrence and development of uveitis.

Chemokines are a class of proinflammatory cytokines that are able to attract and activate the migration of circulating leukocytes under both physiological and pathological conditions [7]. According to the related structure and function, four subfamilies of human chemokines are classified: CC chemokines, CXC chemokines, CX3C family, and C family. Previous studies showed that chemokines are involved in various inflammatory and autoimmune diseases [8, 9]. Chemokines also contribute to the pathogenesis of uveitis, and previous researches showed that a higher chemokine production might be responsible for the more severe clinical manifestations in Behcet's disease [10]. A comparison of Japanese VKH disease patients with controls indicated a dramatic decrease in the chemokine* CSF-CCL2/MCP-1* [11].

Genetic variations of chemokine genes have been demonstrated responsible for the induction of chronic inflammation [7].* RANTES (CCL5)* is associated with diabetes mellitus type 1 both genetically and functionally [12]. In the onset and development of childhood Idiopathic Thrombocytopenic Purpura, the polymorphism of* SDF-1 (CXCL12)* gene may be implicated [13]. Intron 1 of the* CXCL9 *gene (rs2276886) polymorphism may be closely related to pediatric Crohn's disease [14]. Among Chinese Han individuals, genetic variations of* CXCL12*-3′G801A are involved in the pathogenesis of systemic lupus erythematosus [15]. Only few studies have analyzed the association of uveitis with chemokine gene polymorphisms. In Caucasian patients with* HLA-B27* associated acute anterior uveitis, the CCL2-2518G allele was found significantly increased [16] and* IL-8 (CXCL8)* gene polymorphisms may affect susceptibility to BD in Turkey [17]. However, the association between other chemokine gene polymorphisms with uveitis is largely unknown and has been addressed recently by our group. Earlier we reported that* CCL2* polymorphisms were protective for BD [18]. In this study, we expanded the amount of chemokines SNPs and also included VKH disease patients. The results show that none of the other chemokine genes polymorphisms showed an association with BD or VKH disease in the Chinese Han population.

## 2. Material and Methods

### 2.1. Study Population

Our study recruited 371 BD and 371 VKH disease patients and 605 healthy individuals which are all from Chinese Han population in the First Affiliated Hospital of Chongqing Medical University from January 2009 to April 2015 (Chongqing, China). According to race (Chinese Han) and geography, patients and the controls were matched. Diagnosis for BD and VKH disease followed the standard of the International Study Group for BD [19] and First International Workshop for VKH disease [20], respectively. The local research ethics committee approved the study and all the recruited individuals signed informed consent before donating blood samples. The Declaration of Helsinki adhered to the tenets.

### 2.2. Single Nucleotide Polymorphism (SNP) Selection

Screening of target chemokine gene SNPs was according to previously published studies which showed a positive association with other autoimmune and inflammatory diseases. Linkage disequilibrium (LD) data from the Han Chinese Hap Map database were taken into account. Twenty-seven SNPs of twelve genes with a minor allele frequency > 0.05 in Han Chinese were selected. These 27 SNPs in 12 chemokine genes, included 4 SNPs (rs1024610, rs1024611, rs13900, and rs4586) of* CCL2* [21, 22], 5 SNPs (rs4251719, rs2306630, rs2107538, rs9355610, and rs2280788) of* CCL5* [12, 23, 24], 1 SNP (rs854680) of* CCL16* [25], 2 SNPs (rs223828 and rs223895) of* CCL17* [26–28], 3 SNPs (rs951005, rs2492358, and rs2812378) of* CCL21* [29–31], 1 SNP (rs4359426) of* CCL22* [32], 2 SNPs (rs2302004 and rs2302005) of* CCL24* [33], 3 SNPs (rs2227306, rs2227543, and rs4694178) of* CXCL8* [34], 2 SNPs (rs2276886 and rs2869460) of* CXCL9* [14, 35], 1 SNP (rs2869462) of* CXCL10* [35], 2 SNPs (rs1801157 and rs2839693) of* CXCL12* [13, 15], and 1 SNP (rs2277680) of* CXCL16* [36]. We excluded rs1024611 of* CCL2*, since a study concerning this gene had been reported previously by our group [18].

### 2.3. DNA Extraction and Genotyping

Peripheral blood of the three experimental groups including BD and VKH disease patients and the controls was subjected to genomic DNA extraction with the QIAmp DNA Blood Mini Kit (Qiagen Inc., Valencia, CA, USA) and the DNA was stored at −80°C. The Applied Biosystems 7500 Real-Time PCR system was utilized to genotype* CCL17*/rs223828 (TagMan assay ID: C_30530263_10) by the TaqMan SNP Genotyping Assay (Applied Biosystems, Foster City, CA, USA). Genotype identification of the other 25 SNPs was conducted with the iPLEX Gold genotyping assay and Sequenom MassARRAY (Sequenom, CA, USA). Sequenom SNP Assay Design software version 3.0 was used to design primers of iPLEX reactions. Primer sequences used were shown in Table 1. The protocol and experimental requirements were performed strictly based on the instructions.

### 2.4. Statistical Analysis

Hardy-Weinberg equilibrium (HWE) analysis was carried out by the Chi-square (*χ*^2^) test in healthy samples while the genotype frequency was estimated by direct counting. No SNP significantly deviated from HWE (*P* > 0.05). Fisher's exact test or *χ*^2^ test was applied to evaluate the differences in allele and genotype frequencies of all SNPs between patients and healthy controls using SPSS (version 17.0; SPSS Inc., Chicago, IL). The Bonferroni method was conducted to perform correction for multiple comparisons whereby the *P* value was multiplied with the number of comparisons (*P* corrected (*Pc*)). It was considered to be significant when *Pc* < 0.05. In those genes having more than one SNP we also performed a haplotype analysis. Haplotypes with a frequency of 0.03 or larger were included in the analysis [37, 38]. *P* values for haplotypes were multiplied with the number of haplotypes in each gene. *Pc* < 0.05 was considered as significant. Gene-gene interaction analysis was performed using MDR software (MDR 3.0.2 obtained from https://sourceforge.net/projects/mdr/).

## 3. Results

### 3.1. Clinical Features

The demographics and clinical symptoms of BD and VKH disease and demographics of controls are all showed in Table 2. The healthy cohort is comprised of 321 men and 284 women, who were on average 38.6 ± 11.1 years old. The BD patients consisted of 371 subjects (326 men and 45 women), 33.2 ± 8.4 years old on average. The VKH disease group contained 371 subjects (204 men and 167 women), and the patients were on average 39.8 ± 13.9 years old.

### 3.2. Chemokine Genotyping Results

Twenty-six SNPs covering 12 chemokine genes (*CCL2*,* CCL5, CCL16, CCL17*,* CCL21, CCL22*,* CCL24*,* CXCL8*,* CXCL9*,* CXCL10, CXCL12*, and* CXCL16*) were genotyped successfully and all SNPs of controls met the Hardy-Weinberg equilibrium. There was no significant difference in allelic and genotypic frequencies for all the 26 SNPs in the patients of BD or VKH disease compared with the controls (Supplemental Tables 1 and 2 in Supplementary Material available online at https://doi.org/10.1155/2017/1274960). However, *P*_uncorr_ values showed a weak association of five SNPs of five genes in BD and three SNPs of three genes in VKH disease (Tables 3 and 4). Compared with BD patients, the frequency of the* CCL5*/rs2107538 CC genotype (*P* = 0.048, OR = 1.309, and 95% CI = 1.002–1.710) was decreased and the* CCL5*/rs2107538 CT genotype frequency (*P* = 0.042, OR = 0.76, and 95% CI = 0.583–0.991) was increased in the controls. A higher frequency of* CCL17*/rs223828 C allele (*P* = 0.028, OR = 1.582, and 95% CI = 1.048–2.389) and a lower TT genotype frequency (*P* = 0.020, OR = 0.795, and 95% CI = 0.655–0.965) were found in the patients of BD. In* CCL22*/rs4359426, the frequency of the AA genotype (*P* = 0.012, OR = 3.071, and 95% CI = 1.227–7.685) was increased in BD patients. The* CXCL10*/rs2869462 showed an increased frequency of the CC genotype and C allele (*P* = 0.034, OR = 1.347, and 95% CI = 1.023–1.774; *P* = 0.006, OR = 1.313, and 95% CI = 1.081–1.595, resp.), and* CXCL12*/rs1801157 showed a decreased frequency of the CT genotype (*P* = 0.047, OR = 0.761, and 95% CI = 0.581–0.996) in BD patients. In VKH disease patients, there was an increased frequency of the* CCL5*/rs9355610 A allele (*P* = 0.029, OR = 0.805, and 95% CI = 0.662–0.979) and* CXCL8*/rs2227543 CT genotype (*P* = 0.016, OR = 0.72, and 95% CI = 0.552–0.940). In* CXCL12*/rs1801157, a weak association was detected in the C allele and CC and CT genotype in VKH disease (*P* = 0.01, OR = 1.327, and 95% CI = 1.069–1.647; *P* = 0.00118, OR = 1.556, and 95% CI = 1.190–2.033; *P* = 8.463 × 10^−4^, OR = 0.627, and 95% CI = 0.476–0.826). However, after correction for multiple comparisons, all associations described above lost statistical significance.

### 3.3. Haplotype Analysis

The haplotypes of chemokine genes* (CCL2, CCL5, CCL17, CCL21, CCL24, CXCL8, CXCL9, *and* CXCL12)* having more than one SNP were analyzed using the website http://analysis.bio-x.cn/myAnalysis.php. The haplotype TC of the* CXCL12* gene including two SNPs (rs1801157 and rs2839693) showed a significant association with VKH (*P* = 0.008, *Pc* = 0.032, OR = 0.745, and 95% CI = 0.599–0.927) (Table 5) compared with healthy controls. The other tested haplotypes failed to show an association with either BD or VKH.

### 3.4. Stratified Analysis according to Gender and Main Clinical Manifestations of BD and VKH Disease

Stratified analyses were conducted to investigate whether the 26 SNPs have an association with gender and the primary clinical features in BD and VKH disease. BD in our population is more often seen in males and we therefore believe that a gender analysis might also be involved in the genetic predisposition to this disease and a previous study showed that chemokine gene SNPs of both* CCL2* gene and* CCL5* were more prevalent in males than females with BD [39]. To further confirm whether gender could influence genotype and allele frequencies in both diseases we performed the gender stratified study in these two diseases. We chose clinical manifestations with the frequency of approximately 50%. These included the presence of genital ulcers in BD and sunset glow fundus in VKH disease, respectively. Following Bonferroni correction, no association was observed after stratification by gender (Supplemental Tables 3 and 4). Also no significant differences were detected in these SNPs after stratifying VKH with sunset glow fundus or not. Additionally, no significant association was observed when BD was stratified by genital ulcer. MDR analysis was performed to test the gene-gene (epistatic effect) analysis interaction among 26 SNPs of 12 chemokine genes and this analysis showed that no gene-gene interaction existed in these two diseases. (Supplemental Tables 5 and 6).

## 4. Discussion

In the present study, we described the genotyping of 371 BD and 371 VKH patients and 605 controls from Chinese Han population for twenty-six SNPs in twelve chemokine genes. The AA genotype of MCP-1-2518 (rs1024611/*CCL2*) has been shown by our previous research to have a protective association with BD in the Chinese Han population [18]. Extension of this latter study with other chemokine polymorphisms and including another uveitis entity (VKH) did not reveal any new associations, although haplotype analysis did show that the haplotype TC of the* CXCL12* gene including rs1801157 and rs2839693 shows a significant association with VKH.

Behcet's disease, which is considered an autoinflammatory disorder, is characterized by posterior or generalized uveitis with a chronic nature and with recurrent episodes [2]. VKH disease is considered as a multisystem disorder caused by an autoimmune response against melanocyte associated antigens [3]. The attraction of leukocytes to tissues is an important feature of inflammation and is mediated by the local release of chemokines [40]. Genetic variation in the genes encoding these chemokines may affect their function and may be associated with disease predisposition. Several studies have reported investigations concerning the association of a limited number of chemokine genetic variations in patients with different uveitis entities [21, 39, 41], but a large scale analysis on chemokine gene associations with BD or VKH disease has not been reported.

Despite the fact that the 26 SNPs chosen for our study have been proved to be associated with several other immune-mediated diseases, we did not detect any significant association between these SNPs and the two uveitis entities, BD or VKH disease. An exception is the association of the haplotype TC of the* CXCL12* gene including rs1801157 and rs2839693 with VKH, which suggests that* CXCL12* polymorphisms might be a risk factor contributing to VKH disease in the Chinese population. Our study confirms earlier data presenting the absence of an association between the chemokine genes rs1024610/*CCL2 *and rs2280788/rs2107538/*CCL5* with Behcet's disease or retinal vasculitis in patients from UK [39]. Others showed that the frequency of the T allele of MCP-1 63555 (rs1024610/*CCL2*) was significantly associated with idiopathic anterior uveitis in Caucasian patients [21], which could not be shown in the uveitis entities we studied. This discrepancy may be due to differences in the uveitis entity studied or due to ethnic effects.

Selection of candidate SNPs is a crucial step for a gene variation study. In our study, 26 SNPs covering 12 chemokine genes* (CCL2, CCL5, CCL16, CCL17, CCL21, CCL22, CCL24, CXCL8, CXCL9, CXCL10, CXCL12,* and* CXCL16)* were selected on the basis of earlier association studies in autoimmune diseases, including type 1 diabetes [12], pediatric Crohn's disease [14], and systemic lupus erythematosus [15]. It should be noted that composition and stratification of recruiting population may conclude to different results of an association study. To make sure that our data and results were valid, a series of efforts were made. First of all, the BD patients were diagnosed in strict accordance with the criteria of the International Study Group for BD while the VKH patients were diagnosed in strict accordance with the First International Workshop criteria of VKH disease. Any doubt or uncertainty in patient diagnosis is not allowed. Beyond that, to avoid ethnic bias, BD and VKH disease patients from other ethnic populations other than Chinese Han population were excluded.

Our study has several limitations. We only chose SNPs that have been previously reported to be related to autoimmune and inflammatory diseases, thus other unknown SNPs of chemokine genes with potential association with BD and VKH disease might be excluded. Furthermore, we studied only two common types of uveitis, with all the participants from Chinese Han population. Association of chemokine genes with other types of uveitis or different ethnic populations might also exist and awaits further investigation.

## 5. Conclusions

A large scale analysis of the role of chemokine genes only shows an association of* CCL2* with BD but no effect on predisposition to VKH in Chinese Han population. The haplotype TC of the* CXCL12* gene however did show a significant association with VKH compared with healthy controls.

## Supplementary Material

Supplemental Table 1. Genotype and allele frequencies of chemokine genes polymorphism in BD and healthy cases. Supplemental Table 2. Genotype and allele frequencies of chemokine genes polymorphism in VKH syndrome and healthy cases. Supplemental Table 3. Genotype and allele frequencies of chemokine genes polymorphism in BD stratified by gender. Supplemental Table 4. Genotype and allele frequencies of chemokine genes polymorphism inVKH syndrome stratified by gender. Supplemental Table 5. The best model gene-gene interaction results in BD by MDR. Supplemental Table 6. The best model gene-gene interaction results in VKH by MDR.

## Figures and Tables

**Table 1:** Primers applied in the analysis of restriction fragment length polymorphism (RFLP) in the chemokine genes.

	1st-PCRP	2nd-PCRP	UEP_SEQ
*CCL2*			
rs1024610	ACGTTGGATGTTCTTCCTAGGCCATCTCAC	ACGTTGGATGATTGAATGCGGTCCACCAAG	CATGGTAAAGGATGCACTAAC
rs13900	ACGTTGGATGCTTAAGGCATAATGTTTCAC	ACGTTGGATGCCCAAGAATCTGCAGCTAAC	GGCCATAGCTTTCCCCAGACACC
rs4586	ACGTTGGATGAGCTTCTTTGGGACACTTGC	ACGTTGGATGATGCAATCAATGCCCCAGTC	CTCCCAGTCACCTGCTG
*CCL5*			
rs4251719	ACGTTGGATGTCGTAGGTCCAGACACAAAG	ACGTTGGATGCTTGAGTGATAAAGTGCAGG	AGTGATAAAGTGCAGGTGTTTTA
rs2306630	ACGTTGGATGTCTTTCTGGAACCTTGTGGG	ACGTTGGATGTGTGCCTCAATTGATCCCTG	AAGCTGAAAAACAAGGTTCTCT
rs2107538	ACGTTGGATGACTGTATATCCAGAGGACCC	ACGTTGGATGTGAGTCTTCAAAGTTCCTGC	CTGCTTATTCATTACAGATCTTA
rs9355610	ACGTTGGATGAGGTTAGCACACCAGTAGAG	ACGTTGGATGAATGCACCAGAACGCAAAGC	TGGCTCAGAATACTAGAAATTAG
rs2280788	ACGTTGGATGCTTATGATACCGGCCAATGC	ACGTTGGATGCTCAGGCTGGCCCTTTATAG	GCCCTTTATAGGGCCAGTT
*CCL16*			
rs854680	ACGTTGGATGAAGTCAAACGGTCCAGCAAC	ACGTTGGATGCCACATGATTGAGCTCACAC	GTGAGCTCACACTCTTTC
*CCL17*			
rs223895	ACGTTGGATGCTGGTAATCACTGCCCAATG	ACGTTGGATGATCCTCAGCAATGTGGCTTC	TTGGCTTCCAGCTCTG
*CCL21*			
rs951005	ACGTTGGATGGCTCTTCTTGAGTTTGTGCG	ACGTTGGATGTGGGAAAGGGACCAACCATC	CAAACCTATATAGGACCCACA
rs2492358	ACGTTGGATGGATCTGTTCAGTGTCAAGAG	ACGTTGGATGAGTCCGACATGCTTTTACTG	TTTTACTGTAACTTGTCACTT
rs2812378	ACGTTGGATGCAGGCCCAGACATATTCAAC	ACGTTGGATGCTGAAAGCTGGATTTGCTGG	CCCCACTTATCTCAAACTAA
*CCL22*			
rs4359426	ACGTTGGATGCTATGTCCCTTTGCAGACAC	ACGTTGGATGAGTTGCTTGAAGCGCCACAG	GGCAGTCTGTAGGCGA
rs2302004	ACGTTGGATGAACACCGCCAGTCCATTCAC	ACGTTGGATGATCATCCCTACGGGTAAGAC	CGCTGGGGAAAGGG
rs2302005	ACGTTGGATGTGTTTGGTGCAGGGTTCAAG	ACGTTGGATGACAAAGAACATGCAGCAGGG	CCTAGAAGAGGAGAGAGA
*CXCL8*			
rs2227306	ACGTTGGATGCCCTTGACCTCAGTTAGTTC	ACGTTGGATGGGTATCACAGAGGATTATGC	ACTCTAACTCTTTATATAGGAAGT
rs2227543	ACGTTGGATGCCTGGGCAAACTATGTATGG	ACGTTGGATGATCGTCATTAGGTATCTGCC	AGAAGCAATAGTGAGTTTGTTGTACT
rs4694178	ACGTTGGATGTTTCTGCCACAAAGACATCC	ACGTTGGATGCCTCTCAAGAACAAACTTGG	TCTCAAGAACAAACTTGGAATTAA
*CXCL9*			
rs2276886	ACGTTGGATGGTTCTGCCTGCATTGAAGAG	ACGTTGGATGGTGTTTGACTTGGCAGATCG	TGAGAAGCTTTTATGACTGA
rs2869460	ACGTTGGATGCTGCCCGAATATTACACCTG	ACGTTGGATGAGCGTGGAGTCTCTAAGAAG	GTTGTACACTTGATGTACACTTTA
*CXCL10*			
rs2869462	ACGTTGGATGCACTGTTGTATTACTGAGAC	ACGTTGGATGGAACATTTCCTTGTGTCTAC	TCTCATATTTCTAGCCTCTT
*CXCL12*			
rs1801157	ACGTTGGATGAAGCCTAGTGAAGGCTTCTC	ACGTTGGATGTGCTGCCTCAGCTCAGGGTA	ACAGAAGAGGCAGACC
rs2839693	ACGTTGGATGCTCTGTTGCTCTCCATTCTC	ACGTTGGATGATCAGGAGCAAATACGCCAG	AAGACAGGATGCTCTAGG
*CXCL16*			
rs2277680	ACGTTGGATGGTGGGACCAGCATTTTTTTC	ACGTTGGATGCTCACTCGTCCCAATGAAAC	CCACAGTCTGGCAG

**Table 2:** Clinical features, age, and sex distribution of patients and controls.

Clinical features	Total	%
*Patients with BD*	371	
Mean age ± SD	33.2 ± 8.4	
Male	326	87.9
Female	45	12.1
Uveitis	358	96.5
Oral ulcer	349	94
Genital ulcer	208	56.1
Skin lesion	272	73.3
Arthritis	53	14.3
Pathergy reaction	8	2.2
*Patients with VKH disease*	371	
Mean age ± SD	39.8 ± 13.9	
Male	204	55
Female	167	45
Sunset glow fundus	182	49
Headache	157	42.3
Tinnitus	146	39.4
Vitiligo	123	33.2
Alopecia	136	36.7
Gray hair	58	15.6
*Controls*	605	
Mean age ± SD	38.6 ± 11.1	
Male	321	53.1
Female	284	46.9

BD = Behcet's disease, SD = standard deviation; VKH = Vogt-Koyanagi-Harada.

**Table 3:** Genotype and allele frequencies of five chemokine genes' polymorphism in BD and healthy controls.

Gene	SNP		BD *n* (%)	Controls *n* (%)	*P* value	*Pc* value	OR	95% CI
*CCL5*	*rs2107538*	*Total sample*	*368*	*556*				
		CC	165 (0.448)	213 (0.383)	0.048	NS	1.309	1.002–1.710
		CT	155 (0.421)	272 (0.489)	0.042	NS	0.760	0.583–0.991
		TT	48 (0.130)	71 (0.128)	0.903	NS	1.025	0.692–1.517
		C	485 (0.659)	698 (0.628)	0.170	NS	1.146	0.943–1.393
		T	251 (0.341)	414 (0.372)			0.873	0.718–1.060
*CCL17*	*rs223828 *	*Total sample*	*371*	*604*				
		CC	155 (0.418)	287 (0.575)	0.081	NS	0.793	0.611–1.029
		CT	167 (0.450)	264 (0.437)	0.690	NS	1.054	0.813–1.368
		TT	49 (0.132)	53 (0.088)	0.028	NS	1.582	1.048–2.389
		C	477 (0.643)	838 (0.694)	0.020	NS	0.795	0.655–0.965
		T	265 (0.357)	370 (0.306)				
*CCL22*	*rs4359426*	*Total sample*	*365*	*546*				
		AA	14 (0.038)	7 (0.013)	0.012	NS	3.071	1.227–7.685
		AC	93 (0.255)	134 (0.245)	0.749	NS	1.051	0.774–1.427
		CC	258 (0.707)	405 (0.742)	0.246	NS	0.839	0.624–1.128
		A	121 (0.166)	148 (0.136)	0.075	NS	1.267	0.976–1.645
		C	609 (0.834)	944 (0.864)			0.789	0.608–1.024
*CXCL10*	*rs2869462*	*Total sample*	*360*	*535*				
		CC	149 (0.414)	184 (0.344)	0.034	NS	1.347	1.023–1.774
		CG	160 (0.444)	243 (0.454)	0.773	NS	0.961	0.735–1.258
		GG	51 (0.142)	108 (0.202)	0.210	NS	0.653	0.454–0.939
		C	458 (0.636)	611 (0.571)	0.006	NS	1.313	1.081–1.595
		G	262 (0.364)	459 (0.429)			0.761	0.627–0.925
*CXCL12*	*rs1801157*	*Total sample*	*371*	*547*				
		CC	207 (0.558)	271 (0.495)	0.063	NS	1.285	0.987–1.675
		CT	139 (0.375)	241 (0.441)	0.047	NS	0.761	0.581–0.996
		TT	25 (0.067)	35 (0.064)	0.838	NS	1.057	0.621–1.798
		C	553 (0.745)	783 (0.716)	0.163	NS	1.162	0.941–1.435
		T	189 (0.255)	311 (0.284)			0.860	0.697–1.063

**Table 4:** Genotype and allele frequencies of three chemokine genes' polymorphism in VKH and healthy controls.

Gene	SNP		VKH *n* (%)	Controls *n* (%)	*P* value	*Pc* value	OR	95% CI
*CCL5*	*rs9355610*	*Total sample*	*370*	*555*				
		AA	97 (0.262)	138 (0.249)	0.644	NS	1.074	0.794–1.451
		AG	188 (0.508)	273 (0.492)	0.629	NS	1.067	0.820–1.388
		GG	85 (0.230)	144 (0.259)	0.305	NS	0.851	0.626–1.158
		A	282 (0.516)	549 (0.495)	0.029	NS	0.805	0.662–0.979
		G	358 (0.484)	561 (0.505)			1.242	1.022–1.511
*CXCL8*	*rs2227543*	*Total sample*	*364*	*552*				
		CC	146 (0.401)	187 (0.339)	0.055	NS	1.307	0.994–1.719
		CT	155 (0.426)	280 (0.507)	0.016	NS	0.720	0.552–0.940
		TT	63 (0.173)	85 (0.154)	0.442	NS	1.15	0.805–1.643
		C	447 (0.614)	654 (0.592)	0.355	NS	1.095	0.904–1.326
		T	281 (0.386)	450 (0.408)			0.914	0.754–1.106
*CXCL12*	*rs1801157*	*Total sample*	*368*	*547*				
		CC	223 (0.605)	271 (0.495)	0.001	NS	1.566	1.198–2.048
		CT	122 (0.331)	241 (0.441)	9.443*∗*10^–4^	NS	0.637	0.478–0.829
		TT	23 (0.062)	35 (0.064)	0.928	NS	0.975	0.566–1.679
		C	568 (0.771)	783 (0.716)	0.008	NS	1.343	1.081–1.668
		T	168 (0.228)	311 (0.284)			0.745	0.600–0.925

**Table 5:** Haplotype analysis of *CXCL12* gene polymorphisms in VKH.

Haplotype	VKH (%)	Control (%)	*χ*^2^	*P* value	*Pc* value	OR	95% CI
CC	466.03 (0.633)	630.97 (0.590)	2.875	0.089	NS	1.182	0.974–1.435
CT	104.97 (0.143)	135.03 (0.126)	0.916	0.338	NS	1.143	0.869–1.504
TC	164.97 (0.224)	297.03 (0.278)	6.959	0.008	0.032	0.745	0.599–0.927
TT	0.03 (0.000)	6.97 (0.007)					
