# Supplementary material for: The Association of Chemokine Gene Polymorphisms with VKH and Behcet's Disease in a Chinese Han Population
Source: Biomed Res Int. 2017 May 14;2017:1274960. doi: 10.1155/2017/1274960 (PMC5446887; doi:10.1155/2017/1274960)
Supplement: Supplementary file 1 — Supplemental Table 1. Genotype and allele frequencies of chemokine genes polymorphism in BD and healthy cases. Supplemental Table 2. Genotype and allele frequencies of chemokine genes polymorphism in VKH syndrome and healthy cases. Supplemental Table 3. Genotype and allele frequencies of chemokine genes polymorphism in BD stratified by gender. Supplemental Table 4. Genotype and allele frequencies of chemokine genes polymorphism inVKH syndrome stratified by gender. Supplemental Table 5. The best model gene-gene interaction results in BD by MDR. Supplemental Table 6. The best model gene-gene interaction results in VKH by MDR. [file 1274960.f1.docx]

**Supplemental** **Table 1. Genotype and allele frequencies of chemokine genes polymorphism in BD and healthy cases.**

| Gene | SNP | Genotype | BD n (%) | Controls n (%) | | P value | | Pc value | | OR | | 95%CI |
| --- | --- | --- | --- | --- | --- | --- | --- | --- | --- | --- | --- | --- |
| CCL2 | rs1024610 | total sample | 368 | 555 | |  | |  | |  | |  |
|  |  | AA | 307(0.834) | 473(0.852) | | 0.459 | | NS | | 0.872 | | 0.608-1.252 |
|  |  | AT | 59(0.160) | 80(0.144) | | 0.501 | | NS | | 1.134 | | 0.787-1.634 |
|  |  | TT | 2(0.005) | 2(0.004) | | 0.678 | | NS | | 1.511 | | 0.212 to 10.774 |
|  |  | A | 673(0.914) | 1026(0.924) | | 0.441 | | NS | | 0.875 | | 0.622-1.230 |
|  |  | T | 63(0.086) | 84(0.076) | |  | |  | | 1.143 | | 0.813-1.608 |
|  | rs13900 | total sample | 366 | 547 | |  | |  | |  | |  |
|  |  | CC | 68(0.186) | 102(0.186) | | 0.979 | | NS | | 0.996 | | 0.709-1.399 |
|  |  | CT | 170(0.464) | 269(0.492) | | 0.419 | | NS | | 0.896 | | 0.688-1.169 |
|  |  | TT | 128(0.350) | 176(0.322) | | 0.379 | | NS | | 1.134 | | 0.857-1.500 |
|  |  | C | 306(0.418) | 473(0.432) | | 0.544 | | NS | | 0.943 | | 0.780-1.140 |
|  |  | T | 426(0.582) | 621(0.568) | |  | |  | | 1.06 | | 0.877-1.282 |
|  | rs4586 | total sample | 371 | 605 |  | |  | |  | |  |  |
|  |  | CC | 139(0.375) | 204(0.377) | | 0.234 | | NS | | 1.178 | | 0.900-1.542 |
|  |  | CT | 167(0.450) | 278(0.459) | | 0.775 | | NS | | 0.963 | | 0.743-1.248 |
|  |  | TT | 65(0.175) | 123(0.203) | | 0.28 | | NS | | 0.832 | | 0.597-1.161 |
|  |  | C | 445(0.600) | 686(0.567) | | 0.148 | | NS | | 0.147 | | 0.952-1.381 |
|  |  | T | 297(0.400) | 525(0.433) | |  | |  | |  | |  |
| CCL5 | rs2107538 | total sample | 368 | 556 | |  | |  | |  | |  |
|  |  | CC | 165(0.448) | 213(0.383) | | 0.048 | | NS | | 1.309 | | 1.002-1.710 |
|  |  | CT | 155(0.421) | 272(0.489) | | 0.042 | | NS | | 0.76 | | 0.583-0.991 |
|  |  | TT | 48(0.130) | 71(0.128) | | 0.903 | | NS | | 1.025 | | 0.692-1.517 |
|  |  | C | 485(0.659) | 698(0.628) | | 0.17 | | NS | | 1.146 | | 0.943-1.393 |
|  |  | T | 251(0.341) | 414(0.372) | |  | |  | | 0.873 | | 0.718-1.060 |
|  | rs2306630 | total sample | 366 | 546 | |  | |  | |  | |  |
|  |  | AA | 42(0.115) | 64(0.117) | | 0.909 | | NS | | 0.976 | | 0.645-1.477 |
|  |  | AG | 155(0.423) | 265(0.485) | | 0.066 | | NS | | 0.779 | | 0.597-1.017 |
|  |  | GG | 169(0.462) | 217(0.397) | | 0.054 | | NS | | 1.301 | | 0.995-1.700 |
|  |  | A | 239(0.327) | 393(0.360) | | 0.142 | | NS | | 0.862 | | 0.707-1.051 |
|  |  | G | 493(0.673) | 699(0.640) | |  | |  | | 1.16 | | 0.952-1.413 |
|  | rs9355610 | total sample | 370 | 555 | |  | |  | |  | |  |
|  |  | AA | 87(0.235) | 138(0.249) | | 0.639 | | NS | | 0.929 | | 0.683-1.264 |
|  |  | AG | 198(0.535) | 273(0.492) | | 0.197 | | NS | | 1.189 | | 0.914-1.548 |
|  |  | GG | 85(0.230) | 144(0.259) | | 0.305 | | NS | | 0.851 | | 0.626-1.158 |
|  |  | A | 372(0.503) | 549(0.495) | | 0.733 | | NS | | 1.033 | | 0.585-1.244 |
|  |  | G | 368(0.497) | 561(0.505) | |  | |  | | 0.968 | | 0.804-1.166 |
|  | rs2280788 | total sample | 371 | 601 | |  | |  | |  | |  |
|  |  | CC | 5(0.013) | 6(0.010) | | 0.617 | | NS | | 1.355 | | 0.411-4.471 |
|  |  | CG | 79(0.213) | 132(0.220) | | 0.806 | | NS | | 0.961 | | 0.702-1.317 |
|  |  | GG | 287(0.774) | 463(0.770) | | 0.908 | | NS | | 1.018 | | 0.748-1.387 |
|  |  | C | 89(0.120) | 144(0.220) | | 0.992 | | NS | | 1.001 | | 0.756-1.327 |
|  |  | G | 653(0.880) | 1058(0.880) | |  | |  | |  | |  |
|  | rs4251719 | total sample | 371 | 597 | |  | |  | |  | |  |
|  |  | AA | 42(0.113) | 62(0.104) | | 0.648 | | NS | | 1.102 | | 0.727-1.668 |
|  |  | AG | 152(0.410) | 278(0.466) | | 0.088 | | NS | | 0.796 | | 0.613-1.035 |
|  |  | GG | 177(0.477) | 257(0.430) | | 0.156 | | NS | | 1.207 | | 0.930-1.566 |
|  |  | A | 236(0.318) | 402(0.337) | | 0.397 | | NS | | 0.919 | | 0.756-1.117 |
|  |  | G | 506(0.682) | 792(0.663 | |  | |  | |  | |  |
| CCL16 | rs854680 | total sample | 371 | 557 | |  | |  | |  | |  |
|  |  | AA/TT | 59(0.159) | 88(0.158) | | 0.966 | | NS | | 1.008 | | 0.703-1.444 |
|  |  | AC/TG | 185(0.499) | 285(0.512) | | 0.698 | | NS | | 0.939 | | 0.722-1.221 |
|  |  | CC/GG | 127(0.342) | 184(0.330) | | 0.705 | | NS | | 1.055 | | 0.799-1.393 |
|  |  | A/T | 303(0.408) | 461(0.414) | | 0.815 | | NS | | 0.978 | | 0.809-1.181 |
|  |  | C/G | 439(0.592) | 653(0.586) | |  | |  | | 1.023 | | 0.847-1.235 |
| CCL17 | rs223828 | total sample | 371 | 371 | |  | |  | |  | |  |
|  |  | CC | 155(0.418) | 155(0.418) | | 0.081 | | NS | | 0.793 | | 0.611-1.029 |
|  |  | CT | 167(0.450) | 167(0.450) | | 0.69 | | NS | | 1.054 | | 0.813-1.368 |
|  |  | TT | 49(0.132) | 49(0.132) | | 0.028 | | NS | | 1.582 | | 1.048-2.389 |
|  |  | C | 477(0.643) | 477(0.643) | | 0.02 | | NS | | 0.795 | | 0.655-0.965 |
|  |  | T | 265(0.357) | 265(0.357) | |  | |  | |  | |  |
|  | rs223895 | total sample | 368 | 545 | |  | |  | |  | |  |
|  |  | CC | 99(0.269) | 167(0.306) | | 0.222 | | NS | | 0.833 | | 0.621-1.117 |
|  |  | CT | 190(0.516) | 274(0.503) | | 0.688 | | NS | | 1.056 | | 0.810-1.375 |
|  |  | TT | 79(0.215) | 104(0.191) | | 0.377 | | NS | | 1.159 | | 0.835-1.609 |
|  |  | C | 388(0.527) | 608(0.558) | | 0.197 | | NS | | 0.884 | | 0.733-1.066 |
|  |  | T | 348(0.473) | 482(0.442) | |  | |  | | 1.131 | | 0.938-1.365 |
| CCL21 | rs951005 | total sample | 367 | 548 | |  | |  | |  | |  |
|  |  | AA | 332(0.905) | 487(0.889) | | 0.44 | | NS | | 1.188 | | 0.766-1.842 |
|  |  | AG | 34(0.093) | 59(0.108) | | 0.461 | | NS | | 0.846 | | 0.543-1.320 |
|  |  | GG | 1(0.003) | 2(0.004) | | 1 | | NS | | 0.746 | | 0.067-8.256 |
|  |  | A | 698(0.951) | 1033(0.943) | | 0.434 | | NS | | 1.182 | | 0.776-1.801 |
|  |  | G | 36(0.049) | 63(0.057) | |  | |  | | 0.846 | | 0.555-1.288 |
|  | rs2812378 | total sample | 368 | 555 | |  | |  | |  | |  |
|  |  | AA | 321(0.872) | 494(0.890) | | 0.41 | | NS | | 0.843 | | 0.562-1.265 |
|  |  | AG | 44(0.120) | 57(0.103) | | 0.422 | | NS | | 1.186 | | 0.782-1.801 |
|  |  | GG | 3(0.008) | 4(0.007) | | 1 | | NS | | 1.132 | | 0.252-5.088 |
|  |  | A | 686(0.932) | 1045(0.940) | | 0.414 | | NS | | 0.853 | | 0.583-1.249 |
|  |  | G | 50(0.068) | 65(0.060) | |  | |  | | 1.172 | | 0.8-1.715 |
|  | rs2492358 | total sample | 368 | 556 | |  | |  | |  | |  |
|  |  | CC | 2(0.005) | 4(0.007) | | 1 | | NS | | 0.754 | | 0.137-4.138 |
|  |  | CT | 32(0.087) | 57(0.103) | | 0.433 | | NS | | 0.834 | | 0.529-1.313 |
|  |  | TT | 334(0.908) | 495(0.890) | | 0.396 | | NS | | 1.211 | | 0.778-1.883 |
|  |  | C | 36(0.049) | 65(0.058) | | 0.377 | | NS | | 0.828 | | 0.545-1.259 |
|  |  | T | 700(0.951) | 1047(0.942) | |  | |  | | 1.207 | | 0.795-1.834 |
| CCL22 | rs4359426 | total sample | 365 | 546 | |  | |  | |  | |  |
|  |  | AA | 14(0.038) | 7(0.013) | | 0.012 | | NS | | 3.071 | | 1.227-7.685 |
|  |  | AC | 93(0.255) | 134(0.245) | | 0.749 | | NS | | 1.051 | | 0.774-1.427 |
|  |  | CC | 258(0.707) | 405(0.742) | | 0.246 | | NS | | 0.839 | | 0.624-1.128 |
|  |  | A | 121(0.166) | 148(0.136) | | 0.075 | | NS | | 1.267 | | 0.976-1.645 |
|  |  | C | 609(0.834) | 944(0.864) | |  | |  | | 0.789 | | 0.608-1.024 |
| CCL24 | rs2302004 | total sample | 361 | 547 | |  | |  | |  | |  |
|  |  | AA | 43(0.119) | 49(0.090) | | 0.149 | | NS | | 1.374 | | 0.891-2.119 |
|  |  | AG | 145(0.402) | 233(0.426) | | 0.467 | | NS | | 0.905 | | 0.690-1.185 |
|  |  | GG | 173(0.479) | 265(0.484) | | 0.877 | | NS | | 0.979 | | 0.751-1.278 |
|  |  | A | 231(0.320) | 331(0.303) | | 0.433 | | NS | | 1.084 | | 0.886-1.328 |
|  |  | G | 491(0.680) | 763(0.697) | |  | |  | | 0.922 | | 0.753-1.129 |
|  | rs2302005 | total sample | 370 | 555 | |  | |  | |  | |  |
|  |  | AA | 154(0.416) | 245(0.441) | | 0.448 | | NS | | 0.902 | | 0.691-1.177 |
|  |  | AG | 166(0.449) | 244(0.440) | | 0.787 | | NS | | 1.037 | | 0.796-1.351 |
|  |  | GG | 50(0.135) | 66(0.119) | | 0.466 | | NS | | 1.158 | | 0.781-1.716 |
|  |  | A | 474(0.641) | 734(0.661) | | 0.359 | | NS | | 0.913 | | 0.751-1.109 |
|  |  | G | 266(0.359) | 376(0.339) | |  | |  | | 1.095 | | 0.901-1.331 |
| CXCL8 | rs2227306 | total sample | 368 | 551 | |  | |  | |  | |  |
|  |  | CC | 151(0.410) | 213(0.387) | | 0.471 | | NS | | 1.104 | | 0.843-1.446 |
|  |  | CT | 179(0.486) | 271(0.492) | | 0.872 | | NS | | 0.979 | | 0.752-1.274 |
|  |  | TT | 38(0.103) | 67(0.122) | | 0.392 | | NS | | 0.832 | | 0.546-1.268 |
|  |  | C | 481(0.654) | 697(0.632) | | 0.357 | | NS | | 1.096 | | 0.902-1.332 |
|  |  | T | 255(0.346) | 405(0.368) | |  | |  | | 0.912 | | 0.751-1.109 |
|  | rs2227543 | total sample | 368 | 552 | |  | |  | |  | |  |
|  |  | CC | 136(0.370) | 187(0.339) | | 0.338 | | NS | | 1.144 | | 0.869-1.507 |
|  |  | CT | 189(0.514) | 280(0.507) | | 0.851 | | NS | | 1.026 | | 0.788-1.335 |
|  |  | TT | 43(0.117) | 85(0.154) | | 0.111 | | NS | | 0.727 | | 0.491-1.077 |
|  |  | C | 461(0.626) | 654(0.592) | | 0.144 | | NS | | 1.153 | | 0.952-1.397 |
|  |  | T | 275(0.374) | 450(0.408) | |  | |  | | 0.867 | | 0.716-1.050 |
|  | rs4694178 | total sample | 366 | 538 | |  | |  | |  | |  |
|  |  | AA | 130(0.355) | 174(0.323) | | 0.321 | | NS | | 1.152 | | 0.871-1.525 |
|  |  | AC | 187(0.511) | 275(0.511) | | 0.995 | | NS | | 0.999 | | 0.766-1.303 |
|  |  | CC | 49(0.134) | 89(0.165) | | 0.195 | | NS | | 0.78 | | 0.535-1.137 |
|  |  | A | 447(0.611) | 623(0.579) | | 0.179 | | NS | | 1.14 | | 0.942-1.381 |
|  |  | C | 285(0.389) | 453(0.421) | |  | |  | | 0.877 | | 0.724-1.062 |
| CXCL9 | rs2276886 | total sample | 368 | 555 | |  | |  | |  | |  |
|  |  | CC | 148(0.402) | 197(0.355) | | 0.147 | | NS | | 1.223 | | 0.932-1.604 |
|  |  | CT | 168(0.457) | 258(0.465) | | 0.803 | | NS | | 0.967 | | 0.742-1.260 |
|  |  | TT | 52(0.141) | 100(0.180) | | 0.119 | | NS | | 0.749 | | 0.520-1.078 |
|  |  | C | 464(0.630) | 652(0.587) | | 0.64 | | NS | | 1.198 | | 0.989-1.451 |
|  |  | T | 272(0.370) | 458(0.413) | |  | |  | | 0.835 | | 0.689-1.011 |
|  | rs2869460 | total sample | 367 | 546 | |  | |  | |  | |  |
|  |  | CC | 96(0.262) | 169(0.310) | | 0.118 | | NS | | 0.79 | | 0.588-1.062 |
|  |  | CT | 184(0.501) | 249(0.456) | | 0.179 | | NS | | 1.199 | | 0.920-1.563 |
|  |  | TT | 87(0.237) | 128(0.234) | | 0.927 | | NS | | 1.015 | | 0.743-1.386 |
|  |  | C | 376(0.512) | 587(0.538) | | 0.289 | | NS | | 0.904 | | 0.749-1.090 |
|  |  | T | 358(0.488) | 505(0.462) | |  | |  | | 1.107 | | 0.918-1.335 |
| CXCL10 | rs2869462 | total sample | 360 | 535 | |  | |  | |  | |  |
|  |  | CC | 149(0.414) | 184(0.344) | | 0.034 | | NS | | 1.347 | | 1.023-1.774 |
|  |  | CG | 160(0.444) | 243(0.454) | | 0.773 | | NS | | 0.961 | | 0.735-1.258 |
|  |  | GG | 51(0.142) | 108(0.202) | | 0.21 | | NS | | 0.653 | | 0.454-0.939 |
|  |  | C | 458(0.636) | 611(0.571) | | 0.006 | | NS | | 1.313 | | 1.081-1.595 |
|  |  | G | 262(0.364) | 459(0.429) | |  | |  | | 0.761 | | 0.627-0.925 |
| CXCL12 | rs1801157 | total sample | 371 | 547 | |  | |  | |  | |  |
|  |  | CC | 207(0.558) | 271(0.495) | | 0.063 | | NS | | 1.285 | | 0.987-1.675 |
|  |  | CT | 139(0.375) | 241(0.441) | | 0.047 | | NS | | 0.761 | | 0.581-0.996 |
|  |  | TT | 25(0.067) | 35(0.064) | | 0.838 | | NS | | 1.057 | | 0.621-1.798 |
|  |  | C | 553(0.745) | 783(0.716) | | 0.163 | | NS | | 1.162 | | 0.941-1.435 |
|  |  | T | 189(0.255) | 311(0.284) | |  | |  | | 0.86 | | 0.697-1.063 |
|  | rs2839693 | total sample | 369 | 543 | |  | |  | |  | |  |
|  |  | CC | 288(0.780) | 398(0.733) | | 0.103 | | NS | | 1.295 | | 0.949-1.768 |
|  |  | CT | 80(0.217) | 140(0.258) | | 0.155 | | NS | | 0.797 | | 0.582-1.090 |
|  |  | TT | 1(0.003) | 5(0.009) | | 0.41 | | NS | | 0.292 | | 0.034-2.513 |
|  |  | C | 656(0.889) | 936(0.862) | | 0.089 | | NS | | 1.282 | | 0.962-1.708 |
|  |  | T | 82(0.111) | 150(0.138) | |  | |  | | 0.78 | | 0.585-1.039 |
| CXCL16 | rs2277680 | total sample | 364 | 541 | |  | |  | |  | |  |
|  |  | AA | 155(0.426) | 200(0.370) | | 0.096 | | NS | | 1.26 | | 0.964-1.659 |
|  |  | AG | 163(0.448) | 275(0.508) | | 0.074 | | NS | | 0.784 | | 0.601-1024 |
|  |  | GG | 46(0.126) | 66(0.122) | | 0.845 | | NS | | 1.041 | | 0.696-1.557 |
|  |  | A | 473(0.650) | 675(0.624) | | 0.262 | | NS | | 1.118 | | 0.920-1.360 |
|  |  | G | 255(0.350) | 407(0.376) | |  | |  | | 0.894 | | 0.735-1.087 |

**Supplemental Table 2. Genotype and allele frequencies of chemokine genes polymorphism in VKH syndrome and healthy cases.**

| Gene | SNP | Genotype | VKH n (%) | Controls n (%) | | P value | | Pc value | | OR | | 95%CI | |
| --- | --- | --- | --- | --- | --- | --- | --- | --- | --- | --- | --- | --- | --- |
| CCL2 | rs1024610 | total sample | 366 | 555 | |  | |  | |  | |  | |
|  |  | AA | 315(0.861) | 473(0.852) | | 0.723 | | NS | | 1.071 | | 0.734-1.562 | |
|  |  | AT | 47(0.128) | 80(0.144) | | 0.498 | | NS | | 0.875 | | 0.594-1.288 | |
|  |  | TT | 4(0.011) | 2(0.004) | | 0.222 | | NS | | 3.055 | | 0.557-16.767 | |
|  |  | A | 677(0.922) | 1026(0.924) | | 0.966 | | NS | | 1.008 | | 0.708-1.435 | |
|  |  | T | 57(0.078) | 84(0.076) | |  | |  | | 0.992 | | 0.697-1.413 | |
|  | rs13900 | total sample | 370 | 547 | |  | |  | |  | |  | |
|  |  | CC | 67(0.181) | 102(0.186) | | 0.836 | | NS | | 0.965 | | 0.686-1.357 | |
|  |  | CT | 184(0.497) | 269(0.492) | | 0.87 | | NS | | 1.022 | | 0.785-1.331 | |
|  |  | TT | 119(0.322) | 176(0.322) | | 0.997 | | NS | | 0.999 | | 0.753-1.326 | |
|  |  | C | 318(0.430) | 473(0.432) | | 0.911 | | NS | | 0.989 | | 0.819-1.194 | |
|  |  | T | 422(0.570) | 621(0.568) | |  | |  | | 1.011 | | 0.837-1.220 | |
|  | rs4586 | total sample | 351 | 605 |  | |  | |  | |  | |  |
|  |  | CC | 118(0.336) | 204(0.377) | | 0.975 | | NS | | 0.995 | | 0.754-1.315 | |
|  |  | CT | 162(0.461) | 278(0.459) | | 0.951 | | NS | | 1.008 | | 0.774-1.313 | |
|  |  | TT | 71(0.202) | 123(0.203) | | 0.97 | | NS | | 0.994 | | 0.716-1.378 | |
|  |  | C | 398(0.567) | 686(0.567) | | 0.984 | | NS | | 1.002 | | 0.831-1.209 | |
|  |  | T | 304(0.433) | 525(0.433) | |  | |  | |  | |  | |
| CCL5 | rs2107538 | total sample | 369 | 556 | |  | |  | |  | |  | |
|  |  | CC | 151(0.409) | 213(0.383) | | 0.426 | | NS | | 1.115 | | 0.852-1.460 | |
|  |  | CT | 171(0.463) | 272(0.489) | | 0.442 | | NS | | 0.902 | | 0.693-1.174 | |
|  |  | TT | 47(0.127) | 71(0.128) | | 0.988 | | NS | | 0.997 | | 0.672-1.479 | |
|  |  | C | 473(0.641) | 698(0.628) | | 0.563 | | NS | | 1.059 | | 0.873-1.285 | |
|  |  | T | 265(0.359) | 414(0.372) | |  | |  | | 0.945 | | 0.778-1.146 | |
|  | rs2306630 | total sample | 369 | 546 | |  | |  | |  | |  | |
|  |  | AA | 43(0.117) | 64(0.117) | | 0.975 | | NS | | 0.993 | | 0.658-1.499 | |
|  |  | AG | 172(0.466) | 265(0.485) | | 0.568 | | NS | | 0.926 | | 0.711-1.206 | |
|  |  | GG | 154(0.417) | 217(0.397) | | 0.547 | | NS | | 1.086 | | 0.830-1.421 | |
|  |  | A | 258(0.350) | 393(0.360) | | 0.652 | | NS | | 0.956 | | 0.786-1.162 | |
|  |  | G | 480(0.650) | 699(0.640) | |  | |  | | 1.046 | | 0.86-1.272 | |
|  | rs9355610 | total sample | 370 | 555 | |  | |  | |  | |  | |
|  |  | AA | 97(0.262) | 138(0.249) | | 0.644 | | NS | | 1.074 | | 0.794-1.451 | |
|  |  | AG | 188(0.508) | 273(0.492) | | 0.629 | | NS | | 1.067 | | 0.820-1.388 | |
|  |  | GG | 85(0.230) | 144(0.259) | | 0.305 | | NS | | 0.851 | | 0.626-1.158 | |
|  |  | A | 282(0.516) | 549(0.495) | | 0.029 | | NS | | 0.805 | | 0.662-0.979 | |
|  |  | G | 358(0.484) | 561(0.505) | |  | |  | | 1.242 | | 1.022-1.511 | |
|  | rs2280788 | total sample | 354 | 601 | |  | |  | |  | |  | |
|  |  | CC | 9(0.025) | 6(0.010) | | *0.064* | | NS | | 2.587 | | 0.913-7.330 | |
|  |  | CG | 76(0.214) | 132(0.220) | | *0.858* | | NS | | 0.971 | | 0.706-1.336 | |
|  |  | GG | 269(0.759) | 463(0.770) | | 0.711 | | NS | | 0.943 | | 0.692-1.285 | |
|  |  | C | 94(0.133) | 144(0.220) | | 0.407 | | NS | | 1.125 | | 0.852-1.486 | |
|  |  | G | 614(0.867) | 1058(0.880) | |  | |  | |  | |  | |
|  | rs4251719 | total sample | 354 | 597 | |  | |  | |  | |  | |
|  |  | AA | 48(0.135) | 62(0.104) | | 0.139 | | NS | | 1.354 | | 0.905-2.024 | |
|  |  | AG | 161(0.455) | 278(0.466) | | 0.745 | | NS | | 0.957 | | 0.735-1.246 | |
|  |  | GG | 145(0.410) | 257(0.430) | | 0.529 | | NS | | 0.918 | | 0.703-1.198 | |
|  |  | A | 257(0.363) | 402(0.337) | | 0.244 | | NS | | 1.123 | | 0.924-1.364 | |
|  |  | G | 451(0.637) | 792(0.663) | |  | |  | |  | |  | |
| CCL16 | rs854680 | total sample | 370 | 557 | |  | |  | |  | |  | |
|  |  | AA/TT | 49(0.132) | 88(0.158) | | 0.283 | | NS | | 0.814 | | 0.558-1.186 | |
|  |  | AC/TG | 181(0.489) | 285(0.512) | | 0.503 | | NS | | 0.914 | | 0.703-1.189 | |
|  |  | CC/GG | 140(0.378) | 184(0.330) | | 0.133 | | NS | | 1.234 | | 0.938-1.624 | |
|  |  | A/T | 279(0.377) | 461(0.414) | | 0.341 | | NS | | 0.913 | | 0.756-1.102 | |
|  |  | C/G | 461(0.623) | 653(0.586) | |  | |  | | 1.166 | | 0.964-1.411 | |
| CCL17 | rs223828 | total sample | 356 | 604 | |  | |  | |  | |  | |
|  |  | CC | 150(0.421) | 287(0.575) | | 0.106 | | NS | | 0.804 | | 0.618-1.047 | |
|  |  | CT | 167(0.469) | 264(0.437) | | 0.335 | | NS | | 1.138 | | 0.875-1.480 | |
|  |  | TT | 39(0.110) | 53(0.088) | | 0.257 | | NS | | 1.285 | | 0.832-1.984 | |
|  |  | C | 467(0.656) | 838(0.694) | | 0.086 | | NS | | 0.842 | | 0.691-1.025 | |
|  |  | T | 245(0.344) | 370(0.306) | |  | |  | |  | |  | |
|  | rs223895 | total sample | 370 | 545 | |  | |  | |  | |  | |
|  |  | CC | 103(0.278) | 167(0.306) | | 0.361 | | NS | | 0.873 | | 0.653-1.168 | |
|  |  | CT | 191(0.516) | 274(0.503) | | 0.689 | | NS | | 1.055 | | 0.810-1.374 | |
|  |  | TT | 76(0.205) | 104(0.191) | | 0.586 | | NS | | 1.096 | | 0.788-1.526 | |
|  |  | C | 397(0.536) | 608(0.558) | | 0.369 | | NS | | 0.918 | | 0.761-1.107 | |
|  |  | T | 343(0.464) | 482(0.442) | |  | |  | | 1.09 | | 0.903-1.315 | |
| CCL21 | rs951005 | total sample | 370 | 548 | |  | |  | |  | |  | |
|  |  | AA | 340(0.919) | 487(0.889) | | 0.133 | | NS | | 1.42 | | 0.898-2.245 | |
|  |  | AG | 30(0.081) | 59(0.108) | | 0.182 | | NS | | 0.731 | | 0.461-1.159 | |
|  |  | GG | 0(0.00) | 2(0.004) | | - | |  | | - | | - | |
|  |  | A | 710(0.959) | 1033(0.943) | | 0.104 | | NS | | 1.443 | | 0.925-2.253 | |
|  |  | G | 30(0.041) | 63(0.057) | |  | |  | | 0.693 | | 0.444-1.081 | |
|  | rs2812378 | total sample | 369 | 555 | |  | |  | |  | |  | |
|  |  | AA | 332(0.900) | 494(0.890) | | 0.641 | | NS | | 1.108 | | 0.720-1.706 | |
|  |  | AG | 34(0.092) | 57(0.103) | | 0.598 | | NS | | 0.887 | | 0.567-1.386 | |
|  |  | GG | 3(0.008) | 4(0.007) | | 1 | | NS | | 1.129 | | 0.251-5.074 | |
|  |  | A | 698(0.946) | 1045(0.940) | | 0.692 | | NS | | 1.085 | | 0.724-1.628 | |
|  |  | G | 40(0.054) | 65(0.060) | |  | |  | | 0.921 | | 0.614-1.382 | |
|  | rs2492358 | total sample | 368 | 556 | |  | |  | |  | |  | |
|  |  | CC | 0(0.00) | 4(0.007) | | - | |  | |  | |  | |
|  |  | CT | 29(0.079) | 57(0.103) | | 0.225 | | NS | | 0.749 | | 0.469-1.196 | |
|  |  | TT | 339(0.921) | 495(0.890) | | 0.51 | | NS | | 0.84 | | 0.501-1.411 | |
|  |  | C | 29(0.039) | 65(0.058) | | 0.068 | | NS | | 0.661 | | 0.422-1.034 | |
|  |  | T | 707(0.961) | 1047(0.942) | |  | |  | | 1.514 | | 0.967-2.369 | |
| CCL22 | rs4359426 | total sample | 362 | 546 | |  | |  | |  | |  | |
|  |  | AA | 4(0.011) | 7(0.013) | | 1 | | NS | | 0.86 | | 0.250-2.960 | |
|  |  | AC | 106(0.293) | 134(0.245) | | 0.113 | | NS | | 1.273 | | 0.944-1.716 | |
|  |  | CC | 252(0.696) | 405(0.742) | | 0.132 | | NS | | 0.798 | | 0.594-1.071 | |
|  |  | A | 114(0.157) | 148(0.136) | | 0.193 | | NS | | 1.192 | | 0.915-1.553 | |
|  |  | C | 610(0.843) | 944(0.864) | |  | |  | | 0.839 | | 0.644-1.093 | |
| CCL24 | rs2302004 | total sample | 363 | 547 | |  | |  | |  | |  | |
|  |  | AA | 37(0.102) | 49(0.090) | | 0.533 | | NS | | 1.153 | | 0.736-1.807 | |
|  |  | AG | 147(0.405) | 233(0.426) | | 0.529 | | NS | | 0.917 | | 0.700-1.201 | |
|  |  | GG | 179(0.493) | 265(0.484) | | 0.798 | | NS | | 1.035 | | 0.794-1.350 | |
|  |  | A | 221(0.304) | 331(0.303) | | 0.933 | | NS | | 1.009 | | 0.823-1.237 | |
|  |  | G | 505(0.696) | 763(0.697) | |  | |  | | 0.991 | | 0.808-1.216 | |
|  | rs2302005 | total sample | 369 | 555 | |  | |  | |  | |  | |
|  |  | AA | 156(0.423) | 245(0.441) | | 0.575 | | NS | | 0.927 | | 0.710-1.209 | |
|  |  | AG | 172(0.466) | 244(0.440) | | 0.428 | | NS | | 1.113 | | 0.854-1.450 | |
|  |  | GG | 41(0.111) | 66(0.119) | | 0.716 | | NS | | 0.926 | | 0.612-1.401 | |
|  |  | A | 484(0.656) | 734(0.661) | | 0.809 | | NS | | 0.976 | | 0.802-1.188 | |
|  |  | G | 254(0.344) | 376(0.339) | |  | |  | | 1.024 | | 0.842-1.247 | |
| CXCL8 | rs2227306 | total sample | 366 | 551 | |  | |  | |  | |  | |
|  |  | CC | 151(0.413) | 213(0.387) | | 0.431 | | NS | | 1.114 | | 0.851-1.459 | |
|  |  | CT | 158(0.432) | 271(0.492) | | 0.074 | | NS | | 0.785 | | 0.602-1.024 | |
|  |  | TT | 57(0.156) | 67(0.122) | | 0.139 | | NS | | 1.333 | | 0.910-1.950 | |
|  |  | C | 460(0.628) | 697(0.632) | | 0.86 | | NS | | 0.983 | | 0.810-1.193 | |
|  |  | T | 272(0.372) | 405(0.368) | |  | |  | | 1.018 | | 0.839-1.235 | |
|  | rs2227543 | total sample | 364 | 552 | |  | |  | |  | |  | |
|  |  | CC | 146(0.401) | 187(0.339) | | 0.055 | | NS | | 1.307 | | 0.994-1.719 | |
|  |  | CT | 155(0.426) | 280(0.507) | | 0.016 | | NS | | 0.72 | | 0.552-0.940 | |
|  |  | TT | 63(0.173) | 85(0.154) | | 0.442 | | NS | | 1.15 | | 0.805-1.643 | |
|  |  | C | 447(0.614) | 654(0.592) | | 0.355 | | NS | | 1.095 | | 0.904-1.326 | |
|  |  | T | 281(0.386) | 450(0.408) | |  | |  | | 0.914 | | 0.754-1.106 | |
|  | rs4694178 | total sample | 365 | 538 | |  | |  | |  | |  | |
|  |  | AA | 129(0.353) | 174(0.323) | | 0.349 | | NS | | 1.143 | | 0.864-1.514 | |
|  |  | AC | 164(0.449) | 275(0.511) | | 0.068 | | NS | | 0.78 | | 0.598-1.019 | |
|  |  | CC | 72(0.197) | 89(0.165) | | 0.22 | | NS | | 1.24 | | 0.879-1.748 | |
|  |  | A | 422(0.578) | 623(0.579) | | 0.969 | | NS | | 0.996 | | 0.824-1.205 | |
|  |  | C | 308(0.422) | 453(0.421) | |  | |  | | 1.004 | | 0.830-1.214 | |
| CXCL9 | rs2276886 | total sample | 369 | 555 | |  | |  | |  | |  | |
|  |  | CC | 135(0.366) | 197(0.355) | | 0.735 | | NS | | 1.048 | | 0.797-1.379 | |
|  |  | CT | 167(0.453) | 258(0.465) | | 0.714 | | NS | | 0.952 | | 0.731-1.240 | |
|  |  | TT | 67(0.182) | 100(0.180) | | 0.957 | | NS | | 1.009 | | 0.717-1.421 | |
|  |  | C | 437(0.592) | 652(0.587) | | 0.839 | | NS | | 1.02 | | 0.844-1.232 | |
|  |  | T | 301(0.408) | 458(0.413) | |  | |  | | 0.981 | | 0.811-1.185 | |
|  | rs2869460 | total sample | 357 | 546 | |  | |  | |  | |  | |
|  |  | CC | 104(0.291) | 169(0.310) | | 0.56 | | NS | | 0.917 | | 0.685-1.227 | |
|  |  | CT | 160(0.448) | 249(0.456) | | 0.816 | | NS | | 0.969 | | 0.741-1.267 | |
|  |  | TT | 93(0.261) | 128(0.234) | | 0.373 | | NS | | 1.15 | | 0.845-1.566 | |
|  |  | C | 368(0.515) | 587(0.538) | | 0.357 | | NS | | 0.915 | | 0.757-1.105 | |
|  |  | T | 346(0.485) | 505(0.462) | |  | |  | | 1.093 | | 0.905-1.320 | |
| CXCL10 | rs2869462 | total sample | 368 | 535 | |  | |  | |  | |  | |
|  |  | CC | 135(0.367) | 184(0.344) | | 0.479 | | NS | | 1.105 | | 0.838-1.458 | |
|  |  | CG | 166(0.451) | 243(0.454) | | 0.926 | | NS | | 0.987 | | 0.756-1.289 | |
|  |  | GG | 67(0.182) | 108(0.202) | | 0.459 | | NS | | 0.88 | | 0.627-1.235 | |
|  |  | C | 436(0.592) | 611(0.571) | | 0.366 | | NS | | 1.092 | | 0.902-1.321 | |
|  |  | G | 300(0.408) | 459(0.429) | |  | |  | | 0.916 | | 0.757-1.108 | |
| CXCL12 | rs1801157 | total sample | 368 | 547 | |  | |  | |  | |  | |
|  |  | CC | 223(0.605) | 271(0.495) | | 0.001 | | NS | | 1.566 | | 1.198-2.048 | |
|  |  | CT | 122(0.331) | 241(0.441) | | 9.443*10^-4^ | | NS | | 0.637 | | 0.478-0.829 | |
|  |  | TT | 23(0.062) | 35(0.064) | | 0.928 | | NS | | 0.975 | | 0.566-1.679 | |
|  |  | C | 568(0.771) | 783(0.716) | | 0.008 | | NS | | 1.343 | | 1.081-1.668 | |
|  |  | T | 168(0.228) | 311(0.284) | |  | |  | | 0.745 | | 0.600-0.925 | |
|  | rs2839693 | total sample | 361 | 543 | |  | |  | |  | |  | |
|  |  | CC | 268(0.742) | 398(0.733) | | 0.753 | | NS | | 1.05 | | 0.775-1.421 | |
|  |  | CT | 91(0.252) | 140(0.258) | | 0.846 | | NS | | 0.97 | | 0.715-1.317 | |
|  |  | TT | 2(0.006) | 5(0.009) | | 0.709 | | NS | | 0.599 | | 0.116-3.107 | |
|  |  | C | 627(0.868) | 936(0.862) | | 0.691 | | NS | | 1.058 | | 0.802-1.394 | |
|  |  | T | 95(0.132) | 150(0.138) | |  | |  | | 0.945 | | 0.717-1.246 | |
| CXCL16 | rs2277680 | total sample | 369 | 541 | |  | |  | |  | |  | |
|  |  | AA | 154(0.417) | 200(0.370) | | 0.148 | | NS | | 1.221 | | 0.932-1.601 | |
|  |  | AG | 178(0.482) | 275(0.508) | | 0.442 | | NS | | 0.901 | | 0.692-1.175 | |
|  |  | GG | 37(0.100) | 66(0.122) | | 0.31 | | NS | | 0.802 | | 0.524-1.228 | |
|  |  | A | 486(0.659) | 675(0.624) | | 0.131 | | NS | | 1.163 | | 0.956-1.414 | |
|  |  | G | 252(0.341) | 407(0.376) | |  | |  | | 0.86 | | 0.707-1.046 | |

**Supplemental Table 3. Genotype and allele frequencies of chemokine genes polymorphism in BD stratified by gender.**

| Gene | SNP | Genotype | BD n (%) | Controls n (%) | P value | Pc value | OR | 95%CI |
| --- | --- | --- | --- | --- | --- | --- | --- | --- |
| CCL2 | rs1024610 | F | 45 | 260 |  |  |  |  |
|  |  | AA | 36(0.800) | 223(0.858) | 0.318 | NS | 0.664 | 0.296-1.490 |
|  |  | AT | 9(0.200) | 36(0.138) | 0.282 | NS | 1.556 | 0.691-3.500 |
|  |  | TT | 0(0.000) | 1(0.004) | 0.677 | NS | 1.004 | 0.996-1.011 |
|  |  | A | 81(0.9) | 482(0.927) | 0.377 | NS | 0.71 | 0.331-1.523 |
|  |  | T | 9(0.1) | 38(0.073) |  |  | 1.409 | 0.657-3.025 |
|  |  | M | 323 | 295 |  |  |  |  |
|  |  | AA | 270(0.836) | 250(0.847) | 0.695 | NS | 0.917 | 0.595-1.414 |
|  |  | AT | 51(0.158) | 44(0.149) | 0.763 | NS | 1.07 | 0.690-1.658 |
|  |  | TT | 2(0.006) | 1(0.003) | 1 | NS | 1.832 | 0.165-20.307 |
|  |  | A | 591(0.915) | 544(0.922) | 0.646 | NS | 0.909 | 0.604-1.367 |
|  |  | T | 55(0.085) | 46(0.078) |  |  | 1.101 | 0.731-1.656 |
|  | rs13900 | F | 44 | 259 |  |  |  |  |
|  |  | CC | 9(0.205) | 48(0.185) | 0.763 | NS | 1.13 | 0.510-2.508 |
|  |  | CT | 20(0.455) | 136(0.525) | 0.387 | NS | 0.754 | 0.397-1.432 |
|  |  | TT | 15(0.341) | 75(0.290) | 0.491 | NS | 1.269 | 0.644-2.502 |
|  |  | C | 38(0.432) | 232(0.448) | 0.779 | NS | 0.937 | 0.594-1.478 |
|  |  | T | 50(0.568) | 286(0.552) |  |  | 1.067 | 0.677-1.684 |
|  |  | M | 322 | 288 |  |  |  |  |
|  |  | CC | 59(0.183) | 54(0.188) | 0.892 | NS | 0.972 | 0.646-1.463 |
|  |  | CT | 150(0.466) | 133(0.462) | 0.921 | NS | 1.016 | 0.739-1.398 |
|  |  | TT | 113(0.351) | 101(0.351) | 0.995 | NS | 1.001 | 0.717-1.397 |
|  |  | C | 268(0.416) | 241(0.418) | 0.936 | NS | 0.991 | 0.789-1.244 |
|  |  | T | 376(0.584) | 335(0.582) |  |  | 1.009 | 0.804-1.268 |
|  | rs4586 | F | 45 | 284 |  |  |  |  |
|  |  | CC | 16(0.356) | 100(0.352) | 0.964 | NS | 1.015 | 0.526-1.959 |
|  |  | CT | 20(0.444) | 127(0.447) | 0.973 | NS | 0.989 | 0.525-1.862 |
|  |  | TT | 9(0.200) | 57(0.201) | 0.991 | NS | 0.996 | 0.454-2.185 |
|  |  | C | 52(0.578) | 327(0.576) | 0.97 | NS | 1.009 | 0.643-1.582 |
|  |  | T | 38(0.422) | 241(0.424) |  |  |  |  |
|  |  | M | 326 | 321 |  |  |  |  |
|  |  | CC | 123(0.377) | 104(0.324) | 0.155 | NS | 1.264 | 0.915-1.748 |
|  |  | CT | 147(0.451) | 151(0.470) | 0.619 | NS | 0.925 | 0.679-1.260 |
|  |  | TT | 56(0.172) | 66(0.206) | 0.271 | NS | 0.801 | 0.540-1.190 |
|  |  | C | 393(0.603) | 359(0.559) | 0.112 | NS | 1.196 | 0.956-1.492 |
|  |  | T | 259(0.397) | 283(0.441) |  |  |  |  |
| CCL5 | rs2107538 | F | 44 | 261 |  |  |  |  |
|  |  | CC | 22(0.5) | 99(0.379) | 0.13 | NS | 1.636 | 0.861-3.109 |
|  |  | CT | 15(0.341) | 127(0.487) | 0.073 | NS | 0.546 | 0.280-1.065 |
|  |  | TT | 7(0.159) | 35(0.134) | 0.656 | NS | 1.222 | 0.505-2.954 |
|  |  | C | 485(0.659) | 698(0.628) | 0.39 | NS | 1.233 | 0.764-1.990 |
|  |  | T | 251(0.341) | 414(0.372) |  |  | 0.811 | 0.503-1.308 |
|  |  | M | 324 | 295 |  |  |  |  |
|  |  | CC | 143(0.441) | 114(0.396) | 0.166 | NS | 1.254 | 0.910-1.729 |
|  |  | CT | 140(0.432) | 145(0.492) | 0.138 | NS | 0.787 | 0.573-1.081 |
|  |  | TT | 41(0.127) | 36(0.122) | 0.865 | NS | 1.042 | 0.646-1.682 |
|  |  | C | 426(0.657) | 373(0.632) | 0.355 | NS | 1.116 | 0.884-1.409 |
|  |  | T | 222(0.343) | 217(0.368) |  |  | 0.896 | 0.710-1.131 |
|  | rs2306630 | F | 44 | 260 |  |  |  |  |
|  |  | AA | 7(0.159) | 35(0.135) | 0.663 | NS | 1.216 | 0.503-2.941 |
|  |  | AG | 15(0.341) | 124(0.477) | 0.094 | NS | 0.567 | 0.291-1.108 |
|  |  | GG | 22(0.500) | 101(0.388) | 0.163 | NS | 1.574 | 0.829-2.990 |
|  |  | A | 29(0.330) | 194(0.373) | 0.433 | NS | 0.826 | 0.512-1.333 |
|  |  | G | 59(0.670) | 326(0.627) |  |  |  |  |
|  |  | M | 322 | 286 |  |  |  |  |
|  |  | AA | 35(0.109) | 29(0.101) | 0.77 | NS | 1.081 | 0.642-1.818 |
|  |  | AG | 140(0.435) | 141(0.493) | 0.151 | NS | 0.791 | 0.575-1.089 |
|  |  | GG | 147(0.457) | 116(0.406) | 0.206 | NS | 1.231 | 0.892-1.699 |
|  |  | A | 210(0.326) | 199(0.348) | 0.422 | NS | 0.907 | 0.715-1.151 |
|  |  | G | 434(0.674) | 373(0.652) |  |  | 1.103 | 0.869-1.399 |
|  | rs9355610 | F | 45 | 261 |  |  |  |  |
|  |  | AA | 8(0.178) | 69(0.264) | 0.216 | NS | 0.602 | 0.267-1.355 |
|  |  | AG | 26(0.578) | 129(0.494) | 0.301 | NS | 1.4 | 0.739-2.654 |
|  |  | GG | 11(0.244) | 63(0.241) | 0.965 | NS | 1.017 | 0.487-2.124 |
|  |  | A | 42(0.467 | 267(0.511) | 0.432 | NS | 0.836 | 0.534-1.308 |
|  |  | G | 48(0.533) | 255(0.489) |  |  | 1.197 | 0.764-1.873 |
|  |  | M | 325 | 294 |  |  |  |  |
|  |  | AA | 79(0.243) | 69(0.235) | 0.807 | NS | 1.047 | 0.723-1.516 |
|  |  | AG | 172(0.529) | 144(0.490) | 0.327 | NS | 1.171 | 0.854-1.606 |
|  |  | GG | 74(0.228) | 81(0.276) | 0.17 | NS | 0.775 | 0.539-1.116 |
|  |  | A | 330(0.508) | 282(0.480) | 0.323 | NS | 1.119 | 0.895-1.399 |
|  |  | G | 320(0.492) | 306(0.520) |  |  | 0.894 | 0.715-1.117 |
|  | rs2280788 | F | 45 | 284 |  |  |  |  |
|  |  | CC | 1(0.022) | 2(0.007) | 0.32 | NS | 3.205 | 0.285-36.088 |
|  |  | CG | 11(0.244) | 65(0.229) | 0.818 | NS | 1.09 | 0.523-2.271 |
|  |  | GG | 33(0.733) | 217(0.764) | 0.654 | NS | 0.849 | 0.415-1.736 |
|  |  | C | 13(0.144) | 69(0.121) | 0.54 | NS | 1.221 | 0.644-2.314 |
|  |  | G | 77(0.856) | 499(0.879) |  |  |  |  |
|  |  | M | 326 | 317 |  |  |  |  |
|  |  | CC | 4(0.012) | 4(0.013) | 0.968 | NS | 0.972 | 0.241-3.921 |
|  |  | CG | 68(0.209) | 67(0.211) | 0.931 | NS | 0.983 | 0.673-1.437 |
|  |  | GG | 254(0.779) | 246(0.776) | 0.924 | NS | 1.018 | 0.702-1.477 |
|  |  | C | 76(0.117) | 75(0.118) | 0.923 | NS | 0.983 | 0.700-1.381 |
|  |  | G | 576(0.883) | 559(0.882) |  |  |  |  |
|  | rs4251719 | F | 45 | 284 |  |  |  |  |
|  |  | AA | 8(0.178) | 34(0.119) | 0.278 | NS | 1.59 | 0.684-3.697 |
|  |  | AG | 14(0.311) | 128(0.451) | 0.079 | NS | 0.55 | 0.281-1.079 |
|  |  | GG | 23(0.511) | 122(0.430) | 0.306 | NS | 1.388 | 0.739-2.606 |
|  |  | A | 30(0.333) | 196(0.345) | 0.828 | NS | 0.949 | 0.592-1.520 |
|  |  | G | 60(0.666) | 372(0.655) |  |  |  |  |
|  |  | M | 326 | 313 |  |  |  |  |
|  |  | AA | 34(0.104) | 28(0.09) | 0.526 | NS | 1.185 | 0.700-2.006 |
|  |  | AG | 138(0.423) | 150(0.479) | 0.156 | NS | 0.798 | 0.584-1.090 |
|  |  | GG | 154(0.472) | 135(0.431) | 0.297 | NS | 1.181 | 0.864-1.613 |
|  |  | A | 206(0.316) | 206(0.329) | 0.384 | NS | 0.901 | 0.713-1.139 |
|  |  | G | 466(0.684) | 420(0.671) |  |  |  |  |
| CCL16 | rs854680 | F | 45 | 261 |  |  |  |  |
|  |  | AA | 5(0.111) | 40(0.153) | 0.461 | NS | 0.691 | 0.257-1.856 |
|  |  | AC | 19(0.422) | 127(0.487) | 0.425 | NS | 0.771 | 0.407-1.461 |
|  |  | CC | 21(0.467) | 94(0.360) | 0.173 | NS | 1.555 | 0.821-2.942 |
|  |  | A | 29(0.322) | 207(0.397) | 0.181 | NS | 0.723 | 0.450-1.164 |
|  |  | C | 61(0.678) | 315(0.603) |  |  | 1.382 | 0.859-2.224 |
|  |  | M | 326 | 296 |  |  |  |  |
|  |  | AA | 54(0.166) | 48(0.162) | 0.907 | NS | 1.026 | 0.67-1.569 |
|  |  | AC | 166(0.509) | 158(0.534) | 0.54 | NS | 0.906 | 0.661-1.242 |
|  |  | CC | 106(0.325) | 90(0.304) | 0.572 | NS | 1.103 | 0.786-1.548 |
|  |  | A | 274(0.420) | 254(0.429) | 0.754 | NS | 0.965 | 0.770-1.208 |
|  |  | C | 378(0.580) | 338(0.571) |  |  | 1.037 | 0.828-1.298 |
| CCL17 | rs223828 | F | 45 | 284 |  |  |  |  |
|  |  | CC | 19(0.422) | 136(0.479) | 0.479 | NS | 0.795 | 0.421-1.502 |
|  |  | CT | 20(0.444) | 122(0.430) | 0.852 | NS | 1.062 | 0.564-2.001 |
|  |  | TT | 6(0.133) | 26(0.091) | 0.379 | NS | 1.527 | 0.591-3.945 |
|  |  | C | 58(0.644) | 394(0.694) | 0.35 | NS | 0.8 | 0.502-1.277 |
|  |  | T | 32(0.356) | 174(0.306) |  |  |  |  |
|  |  | M | 326 | 320 |  |  |  |  |
|  |  | CC | 136(0.417) | 151(0.472) | 0.162 | NS | 0.801 | 0.587-1.093 |
|  |  | CT | 147(0.451) | 142(0.444) | 0.855 | NS | 1.029 | 0.755-1.404 |
|  |  | TT | 43(0.132) | 27(0.084) | 0.052 | NS | 1.649 | 0.992-2.741 |
|  |  | C | 419(0.643) | 444(0.694) | 0.051 | NS | 0.794 | 0.629-1.001 |
|  |  | T | 233(0.357) | 196(0.306) |  |  |  |  |
|  | rs223895 | F | 44 | 260 |  |  |  |  |
|  |  | CC | 9(0.205) | 76(0.292) | 0.23 | NS | 0.623 | 0.285-1.358 |
|  |  | CT | 27(0.614) | 138(0.531) | 0.308 | NS | 1.404 | 0.730-2.700 |
|  |  | TT | 8(0.182) | 46(0.177) | 0.937 | NS | 1.034 | 0.451-2.370 |
|  |  | C | 45(0.511) | 290(0.558) | 0.419 | NS | 0.83 | 0.528-1.305 |
|  |  | T | 43(0.489) | 230(0.442) |  |  | 1.205 | 0.766-1.894 |
|  |  | M | 324 | 285 |  |  |  |  |
|  |  | CC | 90(0.278) | 91(0.319) | 0.263 | NS | 0.82 | 0.579-1.161 |
|  |  | CT | 163(0.503) | 136(0.477) | 0.524 | NS | 1.109 | 0.807-1.525 |
|  |  | TT | 71(0.219) | 58(0.204) | 0.638 | NS | 1.098 | 0.743-1.623 |
|  |  | C | 343(0.529) | 318(0.558) | 0.318 | NS | 0.891 | 0.711-1.117 |
|  |  | T | 305(0.471) | 252(0.442) |  |  | 1.122 | 0.895-1.407 |
| CCL21 | rs951005 | F | 44 | 260 |  |  |  |  |
|  |  | AA | 38(0.864) | 231(0.888) | 0.633 | NS | 0.795 | 0.309-2.043 |
|  |  | AG | 5(0.114) | 28(0.108) | 0.907 | NS | 1.062 | 0.387-2.917 |
|  |  | GG | 1(0.023) | 1(0.004) | 0.269 | NS | 6.023 | 0.370-98.117 |
|  |  | A | 81(0.920) | 490(0.942) | 0.428 | NS | 0.708 | 0.301-1.667 |
|  |  | G | 7(0.080) | 30(0.058) |  |  | 1.412 | 0.6-3.321 |
|  |  | M | 323 | 288 |  |  |  |  |
|  |  | AA | 294(0.910) | 256(0.889) | 0.38 | NS | 1.267 | 0.746-2.152 |
|  |  | AG | 29(0.090) | 31(0.108) | 0.459 | NS | 0.818 | 0.480-1.394 |
|  |  | GG | 0(0.00) | 1(0.003) | - |  | - | - |
|  |  | A | 617(0.955) | 543(0.943) | 0.324 | NS | 1.293 | 0.775-2.158 |
|  |  | G | 29(0.045) | 33(0.057) |  |  | 0.773 | 0.463-1.291 |
|  | rs2812378 | F | 45 | 261 |  |  |  |  |
|  |  | AA | 40(0.889) | 232(0.892) | 0.946 | NS | 0.966 | 0.352-2.648 |
|  |  | AG | 5(0.111) | 24(0.092) | 0.691 | NS | 1.229 | 0.443-3.409 |
|  |  | GG | 0(0.00) | 5(0.015) | - |  | - | - |
|  |  | A | 85(0.944) | 488(0.938) | 0.826 | NS | 1.115 | 0.422-2.941 |
|  |  | G | 5(0.056) | 32(0.062) |  |  | 0.897 | 0.340-2.367 |
|  |  | M | 323 | 295 |  |  |  |  |
|  |  | AA | 281(0.870) | 262(0.888) | 0.49 | NS | 0.843 | 0.518-1.370 |
|  |  | AG | 39(0.121) | 33(0.112) | 0.731 | NS | 1.09 | 0.666-1.785 |
|  |  | GG | 3(0.009) | 0(0.00) | - |  | - | - |
|  |  | A | 601(0.930) | 557(0.994) | 0.322 | NS | 0.791 | 0.498-1.258 |
|  |  | G | 45(0.070) | 33(0.056) |  |  | 1.264 | 0.795-2.009 |
|  | rs2492358 | F | 45 | 261 |  |  |  |  |
|  |  | CC | 2(0.044) | 2(0.008) | 0.105 | NS | 6.023 | 0.826-43.903 |
|  |  | CT | 4(0.089) | 28(0.107) | 1 | NS | 0.812 | 0.271-2.436 |
|  |  | TT | 39(0.867) | 231(0.885) | 0.724 | NS | 0.844 | 0.330-2.161 |
|  |  | C | 8(0.089) | 32(0.061) | 0.328 | NS | 1.494 | 0.665-3.356 |
|  |  | T | 82(0.911) | 490(0.939) |  |  | 0.669 | 0.298-1.504 |
|  |  | M | 323 | 295 |  |  |  |  |
|  |  | CC | 0(0.00) | 2(0.007) | - |  |  |  |
|  |  | CT | 28(0.087) | 29(0.098) | 0.618 | NS | 0.871 | 0.505-1.502 |
|  |  | TT | 295(0.913) | 264(0.895) | 0.437 | NS | 1.237 | 0.723-2.117 |
|  |  | C | 28(0.043) | 33(0.056) | 0.307 | NS | 0.765 | 0.456-1.282 |
|  |  | T | 618(0.957) | 557(0.944) |  |  | 1.308 | 0.78-2.192 |
| CCL22 | rs4359426 | F | 44 | 260 |  |  |  |  |
|  |  | AA | 1(0.023) | 4(0.015) | 0.545 | NS | 1.488 | 0.162-13.635 |
|  |  | AC | 12(0.273) | 69(0.265) | 0.919 | NS | 1.038 | 0.506-2.129 |
|  |  | CC | 31(0.705) | 187(0.719) | 0.841 | NS | 0.931 | 0.461-1.878 |
|  |  | A | 14(0.159) | 77(0.148) | 0.789 | NS | 1.088 | 0.585-2.024 |
|  |  | C | 74(0.841) | 443(0.852) |  |  | 0.919 | 0.494-1.709 |
|  |  | M | 321 | 286 |  |  |  |  |
|  |  | AA | 13(0.04) | 3(0.01) | 0.023 | NS | 3.982 | 1.123-14.117 |
|  |  | AC | 81(0.252) | 65(0.227) | 0.471 | NS | 1.148 | 0.789-1.668 |
|  |  | CC | 227(0.707) | 218(0.762) | 0.126 | NS | 0.753 | 0.524-1.083 |
|  |  | A | 107(0.167) | 71(0.124) | 0.036 | NS | 1.411 | 1.021-1.951 |
|  |  | C | 535(0.833) | 501(0.876) |  |  | 0.709 | 0.513-0.980 |
| CCL24 | rs2302004 | F | 43 | 260 |  |  |  |  |
|  |  | AA | 4(0.093) | 24(0.092) | 1 | NS | 1.009 | 0.332-3.065 |
|  |  | AG | 15(0.349) | 118(0.454) | 0.199 | NS | 0.645 | 0.329-1.264 |
|  |  | GG | 24(0.558) | 118(0.454) | 0.204 | NS | 1.52 | 0.794-2.910 |
|  |  | A | 23(0.267) | 166(0.319) | 0.337 | NS | 0.779 | 0.467-1.299 |
|  |  | G | 63(0.733) | 354(0.681) |  |  | 1.284 | 0.770-2.143 |
|  |  | M | 318 | 287 |  |  |  |  |
|  |  | AA | 39(0.123) | 25(0.087) | 0.156 | NS | 1.465 | 0.863-2.488 |
|  |  | AG | 130(0.409) | 115(0.401) | 0.839 | NS | 1.034 | 0.747-1.432 |
|  |  | GG | 149(0.469) | 147(0.512) | 0.284 | NS | 0.84 | 0.610-1.156 |
|  |  | A | 208(0.327) | 165(0.287) | 0.136 | NS | 1.205 | 0.943-1.539 |
|  |  | G | 428(0.673) | 409(0.713) |  |  | 0.83 | 0.560-1.061 |
|  | rs2302005 | F | 45 | 262 |  |  |  |  |
|  |  | AA | 21(0.467) | 110(0.423) | 0.585 | NS | 1.193 | 0.632-2.252 |
|  |  | AG | 21(0.467) | 120(0.462) | 0.949 | NS | 1.021 | 0.541-1.925 |
|  |  | GG | 3(0.067) | 30(0.115) | 0.441 | NS | 0.548 | 0.160-1.876 |
|  |  | A | 63(0.700) | 340(0.654) | 0.393 | NS | 1.235 | 0.760-2.008 |
|  |  | G | 27(0.300) | 180(0.346) |  |  | 0.81 | 0.498-1.316 |
|  |  | M | 326 | 296 |  |  |  |  |
|  |  | AA | 133(0.409) | 135(0.458) | 0.224 | NS | 0.821 | 0.597-1.129 |
|  |  | AG | 145(0.446) | 124(0.420) | 0.517 | NS | 1.111 | 0.808-1.527 |
|  |  | GG | 47(0.145) | 36(0.122) | 0.41 | NS | 1.216 | 0.763-1.938 |
|  |  | A | 411(0.632) | 394(0.668) | 0.191 | NS | 0.855 | 0.677-1.081 |
|  |  | G | 239(0.368) | 196(0.332) |  |  | 1.169 | 0.925-1.477 |
| CXCL8 | rs2227306 | F | 45 | 257 |  |  |  |  |
|  |  | CC | 15(0.333) | 100(0.389) | 0.477 | NS | 0.785 | 0.402-1.532 |
|  |  | CT | 25(0.556) | 122(0.475) | 0.317 | NS | 1.383 | 0.732-2.615 |
|  |  | TT | 5(0.111) | 35(0.136) | 0.647 | NS | 0.793 | 0.293-2.146 |
|  |  | C | 55(0.611) | 322(0.626) | 0.782 | NS | 0.937 | 0.592-1.484 |
|  |  | T | 35(0.389) | 192(0.374) |  |  | 1.067 | 0.674-1.690 |
|  |  | M | 323 | 294 |  |  |  |  |
|  |  | CC | 136(0.421) | 113(0.384) | 0.353 | NS | 1.165 | 0.844-1.608 |
|  |  | CT | 154(0.477) | 149(0.507) | 0.456 | NS | 0.887 | 0.646-1.217 |
|  |  | TT | 33(0.102) | 32(0.109) | 0.787 | NS | 0.932 | 0.557-1.558 |
|  |  | C | 426(0.659) | 375(0.638) | 0.425 | NS | 1.1 | 0.870-1.390 |
|  |  | T | 220(0.341) | 213(0.362) |  |  | 0.909 | 0.720-1.149 |
|  | rs2227543 | F | 45 | 259 |  |  |  |  |
|  |  | CC | 14(0.311) | 91(0.351) | 0.6 | NS | 0.834 | 0.422-1.647 |
|  |  | CT | 26(0.578) | 123(0.475) | 0.203 | NS | 1.513 | 0.798-2.869 |
|  |  | TT | 5(0.111) | 45(0.174) | 0.295 | NS | 0.594 | 0.222-1.590 |
|  |  | C | 54(0.6) | 305(0.589) | 0.842 | NS | 1.048 | 0.664-1.654 |
|  |  | T | 36(0.4) | 213(0.411) |  |  | 0.955 | 0.605-1.507 |
|  |  | M | 323 | 293 |  |  |  |  |
|  |  | CC | 122(0.378) | 96(0.328) | 0.194 | NS | 1.246 | 0.894-1.736 |
|  |  | CT | 163(0.505) | 157(0.536) | 0.439 | NS | 0.882 | 0.643-1.211 |
|  |  | TT | 38(0.118) | 40(0.137) | 0.482 | NS | 0.843 | 0.524-1.356 |
|  |  | C | 407(0.630) | 349(0.596) | 0.215 | NS | 1.156 | 0.919-1.455 |
|  |  | T | 239(0.370) | 237(0.404) |  |  | 0.865 | 0.687-1.088 |
|  | rs4694178 | F | 44 | 256 |  |  |  |  |
|  |  | AA | 14(0.318) | 84(0.328) | 0.897 | NS | 0.956 | 0.481-1.897 |
|  |  | AC | 25(0.568) | 122(0.477) | 0.261 | NS | 1.445 | 0.758-2.754 |
|  |  | CC | 5(0.114) | 50(0.195) | 0.196 | NS | 0.528 | 0.198-1.409 |
|  |  | A | 53(0.602) | 290(0.566) | 0.53 | NS | 1.159 | 0.731-1.839 |
|  |  | C | 35(0.398) | 222(0.434) |  |  | 0.863 | 0.544-1.368 |
|  |  | M | 322 | 292 |  |  |  |  |
|  |  | AA | 116(0.360) | 90(0.319) | 0.288 | NS | 1.201 | 0.856-1.685 |
|  |  | AC | 162(0.503) | 153(0.543) | 0.333 | NS | 0.854 | 0.620-1.176 |
|  |  | CC | 44(0.137) | 39(0.138) | 0.953 | NS | 0.986 | 0.620-1.569 |
|  |  | A | 394(0.612) | 333(0.590) | 0.449 | NS | 1.093 | 0.868-1.377 |
|  |  | C | 250(0.388) | 231(0.410) |  |  | 0.915 | 0.726-1.152 |
| CXCL9 | rs2276886 | F | 45 | 260 |  |  |  |  |
|  |  | CC | 17(0.378) | 86(0.331) | 0.538 | NS | 1.228 | 0.638-2.366 |
|  |  | CT | 24(0.533) | 126(0.485) | 0.546 | NS | 1.215 | 0.645-2.292 |
|  |  | TT | 4(0.089) | 48(0.185) | 0.115 | NS | 0.431 | 0.147-1.261 |
|  |  | C | 58(0.644) | 298(0.573) | 0.205 | NS | 1.35 | 0.848-2.150 |
|  |  | T | 32(0.356) | 222(0.427) |  |  | 0.741 | 0.465-1.179 |
|  |  | M | 323 | 295 |  |  |  |  |
|  |  | CC | 131(0.406) | 111(0.376) | 0.456 | NS | 1.131 | 0.818-1.563 |
|  |  | CT | 144(0.446) | 132(0.447) | 0.967 | NS | 0.993 | 0.723-1.365 |
|  |  | TT | 48(0.149) | 52(0.176) | 0.351 | NS | 0.816 | 0.531-1.252 |
|  |  | C | 406(0.628) | 354(0.6) | 0.304 | NS | 1.128 | 0.897-1.418 |
|  |  | T | 240(0.372) | 236(0.4) |  |  | 0.887 | 0.705-1.115 |
|  | rs2869460 | F | 45 | 257 |  |  |  |  |
|  |  | CC | 12(0.267) | 81(0.315) | 0.516 | NS | 0.79 | 0.388-1.609 |
|  |  | CT | 25(0.556) | 126(0.490) | 0.419 | NS | 1.3 | 0.687-2.457 |
|  |  | TT | 8(0.178) | 50(0.195) | 0.792 | NS | 0.895 | 0.393-2.041 |
|  |  | C | 49(0.544) | 288(0.560) | 0.78 | NS | 0.938 | 0.598-1.471 |
|  |  | T | 41(0.456) | 226(0.440) |  |  | 1.066 | 0.68-1.672 |
|  |  | M | 322 | 289 |  |  |  |  |
|  |  | CC | 84(0.261) | 88(0.304) | 0.231 | NS | 0.806 | 0.566-1.147 |
|  |  | CT | 159(0.494) | 123(0.426) | 0.91 | NS | 1.316 | 0.956-1.812 |
|  |  | TT | 79(0.245) | 78(0.270) | 0.488 | NS | 0.879 | 0.612-1.265 |
|  |  | C | 327(0.508) | 299(0.517) | 0.739 | NS | 0.963 | 0.769-1.205 |
|  |  | T | 317(0.492) | 279(0.483) |  |  | 1.039 | 0.830-1.301 |
| CXCL10 | rs2869462 | F | 45 | 255 |  |  |  |  |
|  |  | CC | 18(0.4) | 75(0.294) | 0.157 | NS | 1.6 | 0.832-3.078 |
|  |  | CG | 23(0.511) | 125(0.490) | 0.796 | NS | 1.087 | 0.577-2.05 |
|  |  | GG | 4(0.089) | 55(0.216) | 0.049 | NS | 0.355 | 0.122-1.033 |
|  |  | C | 59(0.656) | 275(0.539) | 0.041 | NS | 1.626 | 1.018-2.598 |
|  |  | G | 31(0.344) | 235(0.461) |  |  | 0.615 | 0.385-0.982 |
|  |  | M | 315 | 280 |  |  |  |  |
|  |  | CC | 131(0.416) | 109(0.389) | 0.509 | NS | 1.117 | 0.804-1.551 |
|  |  | CG | 137(0.435) | 118(0.421) | 0.74 | NS | 1.057 | 0.763-1.463 |
|  |  | GG | 47(0.149) | 53(0.189) | 0.192 | NS | 0.751 | 0.488-1.155 |
|  |  | C | 399(0.633) | 336(0.6) | 0.238 | NS | 1.152 | 0.911-1.455 |
|  |  | G | 231(0.367) | 224(0.4) |  |  | 0.868 | 0.687-1.098 |
| CXCL12 | rs1801157 | F | 45 | 251 |  |  |  |  |
|  |  | CC | 23(0.511) | 120(0.478) | 0.683 | NS | 1.141 | 0.605-2.153 |
|  |  | CT | 17(0.378) | 116(0.462) | 0.295 | NS | 0.707 | 0.368-1.356 |
|  |  | TT | 5(0.111) | 15(0.060) | 0.206 | NS | 1.967 | 0.677-5.712 |
|  |  | C | 63(0.7) | 356(0.709) | 0.86 | NS | 0.957 | 0.586-1.562 |
|  |  | T | 27(0.3) | 146(0.291) |  |  | 1.045 | 0.640-1.706 |
|  |  | M | 326 | 296 |  |  |  |  |
|  |  | CC | 184(0.564) | 151(0.510) | 0.175 | NS | 1.244 | 0.907-1.707 |
|  |  | CT | 122(0.374) | 125(0.422) | 0.221 | NS | 0.818 | 0.593-1.129 |
|  |  | TT | 20(0.061) | 20(0.068) | 0.752 | NS | 0.902 | 0.475-1.712 |
|  |  | C | 490(0.752) | 427(0.721) | 0.226 | NS | 1.169 | 0.908-1.505 |
|  |  | T | 162(0.248) | 165(0.279) |  |  | 0.856 | 0.665-1.102 |
|  | rs2839693 | F | 44 | 258 |  |  |  |  |
|  |  | CC | 37(0.841) | 191(0.740) | 0.152 | NS | 1.854 | 0.789-4.357 |
|  |  | CT | 7(0.159) | 66(0.256) | 0.166 | NS | 0.55 | 0.234-1.294 |
|  |  | TT | 0(0) | 1(0.004) | - |  | - | - |
|  |  | C | 81(0.920) | 448(0.868) | 0.17 | NS | 1.756 | 0.779-3.961 |
|  |  | T | 7(0.080) | 68(0.132) |  |  |  |  |
|  |  | M | 325 | 285 |  |  |  |  |
|  |  | CC | 251(0.772) | 207(0.726) | 0.19 | NS | 1.278 | 0.885-1.846 |
|  |  | CT | 73(0.225) | 74(0.260) | 0.313 | NS | 0.826 | 0.57-1.198 |
|  |  | TT | 1(0.003) | 4(0.014) | 0.191 | NS | 0.217 | 0.024-1.951 |
|  |  | C | 575(0.885) | 488(0.856) | 0.138 | NS | 1.288 | 0.921-1.802 |
|  |  | T | 75(0.115) | 82(0.144) |  |  | 0.776 | 0.555-1.086 |
| CXCL16 | rs2277680 | F | 45 | 262 |  |  |  |  |
|  |  | AA | 13(0.289) | 97(0.370) | 0.293 | NS | 0.691 | 0.346-1.380 |
|  |  | AG | 26(0.578) | 133(0.508) | 0.384 | NS | 1.327 | 0.7-2.515 |
|  |  | GG | 6(0.133) | 32(0.122) | 0.833 | NS | 1.106 | 0.434-2.819 |
|  |  | A | 473(0.650) | 327(0.624) | 0.404 | NS | 0.824 | 0.524-1.298 |
|  |  | G | 255(0.350) | 197(0.376) |  |  | 1.213 | 0.77-1.91 |
|  |  | M | 319 | 279 |  |  |  |  |
|  |  | AA | 142(0.445) | 103(0.368) | 0.055 | NS | 1.379 | 0.993-1.914 |
|  |  | AG | 137(0.429) | 143(0.511) | 0.047 | NS | 0.721 | 0.522-0.996 |
|  |  | GG | 40(0.125) | 34(0.121) | 0.883 | NS | 1.037 | 0.637-1.690 |
|  |  | A | 421(0.660) | 349(0.623) | 0.186 | NS | 1.173 | 0.926-1.486 |
|  |  | G | 217(0.340) | 211(0.377) |  |  | 0.853 | 0.673-1.080 |

**Supplemental Table 4. Genotype and allele frequencies of chemokine genes polymorphism inVKH syndrome stratified by gender.**

| Gene | SNP | Genotype | VKH n (%) | Controls n (%) | P value | Pc value | OR | 95%CI |
| --- | --- | --- | --- | --- | --- | --- | --- | --- |
| CCL2 | rs1024610 | F | 164 | 260 |  |  |  |  |
|  |  | AA | 149(0.909) | 223(0.858) | 0.12 | NS | 1.648 | 0.874-3.109 |
|  |  | AT | 13(0.079) | 36(0.138) | 0.063 | NS | 0.536 | 0.275-1.044 |
|  |  | TT | 2(0.012) | 1(0.004) | 0.318 | NS | 3.198 | 0.288-35.547 |
|  |  | A | 311(0.948) | 482(0.927) | 0.221 | NS | 1.442 | 0.8-2.6 |
|  |  | T | 17(0.052) | 38(0.073) |  |  | 0.692 | 0.385-1.250 |
|  |  | M | 202 | 295 |  |  |  |  |
|  |  | AA | 166(0.822) | 250(0.847) | 0.447 | NS | 0.83 | 0.513-1.342 |
|  |  | AT | 34(0.168) | 44(0.149) | 0.564 | NS | 1.154 | 0.708-1.881 |
|  |  | TT | 2(0.010) | 1(0.003) | 0.357 | NS | 2.94 | 0.265-32.641 |
|  |  | A | 366(0.906) | 544(0.922) | 0.37 | NS | 0.814 | 0.520-1.277 |
|  |  | T | 38(0.094) | 46(0.078) |  |  | 1.228 | 0.783-1.925 |
|  | rs13900 | F | 167 | 259 |  |  |  |  |
|  |  | CC | 26(0.156) | 48(0.185) | 0.431 | NS | 0.811 | 0.481-1.367 |
|  |  | CT | 79(0.473) | 136(0.525) | 0.294 | NS | 0.812 | 0.550-1.199 |
|  |  | TT | 62(0.371) | 75(0.290) | 0.078 | NS | 1.449 | 0.958-2.190 |
|  |  | C | 131(0.392) | 232(0.448) | 0.109 | NS | 0.796 | 0.601-1.052 |
|  |  | T | 203(0.608) | 286(0.552) |  |  | 1.257 | 0.950-1.663 |
|  |  | M | 203 | 288 |  |  |  |  |
|  |  | CC | 41(0.202) | 54(0.188) | 0.689 | NS | 1.097 | 0.697-1.725 |
|  |  | CT | 105(0.517) | 133(0.462) | 0.226 | NS | 1.249 | 0.871-1.789 |
|  |  | TT | 57(0.281) | 101(0.351) | 0.102 | NS | 0.723 | 0.489-1.068 |
|  |  | C | 187(0.461) | 241(0.418) | 0.189 | NS | 1.187 | 0.919-1.533 |
|  |  | T | 219(0.539) | 335(0.582) |  |  | 0.843 | 0.652-1.088 |
|  | rs4586 | F | 157 | 307 |  |  |  |  |
|  |  | CC | 59(0.376) | 108(0.352) | 0.61 | NS | 1.109 | 0.744-1.653 |
|  |  | CT | 71(0.452) | 138(0.450) | 0.956 | NS | 1.011 | 0.687-1.488 |
|  |  | TT | 27(0.171) | 61(0.198) | 0.487 | NS | 0.838 | 0.508-1.382 |
|  |  | C | 189(0.602) | 354(0.571) | 0.458 | NS | 1.111 | 0.842-1.465 |
|  |  | T | 125(0.398) | 260(0.423) |  |  |  |  |
|  |  | M | 194 | 330 |  |  |  |  |
|  |  | CC | 59(0.304) | 107(0.324) | 0.633 | NS | 0.911 | 0.621-1.336 |
|  |  | CT | 91(0.469) | 155(0.469) | 0.989 | NS | 0.997 | 0.699-1.423 |
|  |  | TT | 44(0.227) | 68(0.206) | 0.576 | NS | 1.13 | 0.736-1.736 |
|  |  | C | 209(0.539) | 369(0.559) | 0.521 | NS | 0.921 | 0.716-1.185 |
|  |  | T | 179(0.461) | 291(0.441) |  |  |  |  |
| CCL5 | rs2107538 | F | 166 | 261 |  |  |  |  |
|  |  | CC | 69(0.416) | 99(0.379) | 0.454 | NS | 1.164 | 0.782-1.732 |
|  |  | CT | 75(0.452) | 127(0.487) | 0.483 | NS | 0.87 | 0.589-1.285 |
|  |  | TT | 22(0.133) | 35(0.134) | 0.963 | NS | 0.987 | 0.556-1.749 |
|  |  | C | 213(0.642) | 698(0.628) | 0.576 | NS | 1.085 | 0.815-1.444 |
|  |  | T | 119(0.358) | 414(0.372) |  |  | 0.922 | 0.693-1.227 |
|  |  | M | 203 | 295 |  |  |  |  |
|  |  | CC | 82(0.404) | 114(0.396) | 0.694 | NS | 1.076 | 0.747-1.551 |
|  |  | CT | 96(0.473) | 145(0.492) | 0.683 | NS | 0.928 | 0.649-1.327 |
|  |  | TT | 25(0.123) | 36(0.122) | 0.97 | NS | 1.01 | 0.586-1.742 |
|  |  | C | 260(0.640) | 373(0.632) | 0.792 | NS | 1.036 | 0.797-1.347 |
|  |  | T | 146(0.360) | 217(0.368) |  |  | 0.965 | 0.742-1.255 |
|  | rs2306630 | F | 166 | 260 |  |  |  |  |
|  |  | AA | 22(0.133) | 35(0.135) | 0.951 | NS | 0.982 | 0.554-1.742 |
|  |  | AG | 74(0.446) | 124(0.477) | 0.53 | NS | 0.882 | 0.597-1.304 |
|  |  | GG | 70(0.422) | 101(0.388) | 0.495 | NS | 1.148 | 0.772-1.706 |
|  |  | A | 118(0.355) | 194(0.373) | 0.602 | NS | 0.927 | 0.696-1.234 |
|  |  | G | 214(0.645) | 326(0.627) |  |  | 1.079 | 0.810-1.437 |
|  |  | M | 203 | 286 |  |  |  |  |
|  |  | AA | 21(0.103) | 29(0.101) | 0.941 | NS | 1.023 | 0.565-1.850 |
|  |  | AG | 98(0.483) | 141(0.493) | 0.823 | NS | 0.96 | 0.670-1.376 |
|  |  | GG | 84(0.414) | 116(0.406) | 0.856 | NS | 1.034 | 0.718-1.491 |
|  |  | A | 140(0.345) | 199(0.348) | 0.921 | NS | 0.987 | 0.755-1.289 |
|  |  | G | 266(0.655) | 373(0.652) |  |  | 1.014 | 0.776-1.324 |
|  | rs9355610 | F | 166 | 261 |  |  |  |  |
|  |  | AA | 47(0.283) | 69(0.264) | 0.671 | NS | 1.099 | 0.711-1.699 |
|  |  | AG | 82(0.494) | 129(0.494) | 0.996 | NS | 0.999 | 0.677-1.474 |
|  |  | GG | 37(0.223) | 63(0.241) | 0.66 | NS | 0.901 | 0.568-1.432 |
|  |  | A | 176(0.530) | 267(0.511) | 0.595 | NS | 1.077 | 0.818-1.419 |
|  |  | G | 156(0.470) | 255(0.489) |  |  | 0.928 | 0.705-1.222 |
|  |  | M | 204 | 294 |  |  |  |  |
|  |  | AA | 50(0.245) | 69(0.235) | 0.789 | NS | 1.059 | 0.697-1.608 |
|  |  | AG | 106(0.520) | 144(0.490) | 0.513 | NS | 1.127 | 0.788-1.611 |
|  |  | GG | 48(0.235) | 81(0.276) | 0.314 | NS | 0.809 | 0.536-1.222 |
|  |  | A | 206(0.505) | 282(0.480) | 0.432 | NS | 1.107 | 0.860-1.425 |
|  |  | G | 202(0.495) | 306(0.520) |  |  | 0.904 | 0.702-1.163 |
|  | rs2280788 | F | 160 | 291 |  |  |  |  |
|  |  | CC | 4(0.025) | 2(0.007) | 0.108 | NS | 3.705 | 0.671-20.455 |
|  |  | CG | 40(0.25) | 67(0.230) | 0.637 | NS | 1.114 | 0.711-1.748 |
|  |  | GG | 116(0.725) | 222(0.763) | 0.374 | NS | 0.819 | 0.528-1.272 |
|  |  | C | 48(0.15) | 71(0.122) | 0.234 | NS | 1.27 | 0.856-1.885 |
|  |  | G | 272(0.85) | 511(0.878) |  |  |  |  |
|  |  | M | 194 | 317 |  |  |  |  |
|  |  | CC | 5(0.026) | 4(0.013) | 0.273 | NS | 2.07 | 0.549-7.805 |
|  |  | CG | 36(0.185) | 67(0.211) | 0.481 | NS | 0.85 | 0.541-1.335 |
|  |  | GG | 153(0.789) | 246(0.776) | 0.738 | NS | 1.077 | 0.698-1.663 |
|  |  | C | 46(0.119) | 75(0.118) | 0.99 | NS | 1.002 | 0.678-1.482 |
|  |  | G | 342(0.881) | 559(0.882) |  |  |  |  |
|  | rs4251719 | F | 161 | 286 |  |  |  |  |
|  |  | AA | 24(0.149) | 34(0.119) | 0.362 | NS | 1.298 | 0.740-2.279 |
|  |  | AG | 72(0.447) | 129(0.451) | 0.937 | NS | 0.985 | 0.668-1.452 |
|  |  | GG | 65(0.404) | 123(0.430) | 0.588 | NS | 0.897 | 0.606-1.328 |
|  |  | A | 120(0.373) | 197(0.344) | 0.396 | NS | 1.131 | 0.854-1.503 |
|  |  | G | 202(0.627) | 375(0.656) |  |  |  |  |
|  |  | M | 193 | 313 |  |  |  |  |
|  |  | AA | 24(0.124) | 28(0.09) | 0.209 | NS | 1.445 | 0.811-2.575 |
|  |  | AG | 89(0.461) | 150(0.479) | 0.692 | NS | 0.93 | 0.649-1.332 |
|  |  | GG | 80(0.415) | 135(0.431) | 0.71 | NS | 0.933 | 0.649-1.343 |
|  |  | A | 137(0.355) | 206(0.329) | 0.399 | NS | 1.122 | 0.859-1.465 |
|  |  | G | 249(0.645) | 420(0.671) |  |  |  |  |
| CCL16 | rs854680 | F | 166 | 261 |  |  |  |  |
|  |  | AA | 21(0.127) | 40(0.153) | 0.441 | NS | 0.8 | 0.453-1.412 |
|  |  | AC | 87(0.524) | 127(0.487) | 0.45 | NS | 1.162 | 0.787-1.715 |
|  |  | CC | 58(0.349) | 94(0.360) | 0.821 | NS | 0.954 | 0.635-1.433 |
|  |  | A | 129(0.389) | 207(0.397) | 0.816 | NS | 0.967 | 0.729-1.282 |
|  |  | C | 203(0.611) | 315(0.603) |  |  | 1.034 | 0.780-1.371 |
|  |  | M | 204 | 296 |  |  |  |  |
|  |  | AA | 28(0.137) | 48(0.162) | 0.446 | NS | 0.822 | 0.496-1.361 |
|  |  | AC | 94(0.461) | 158(0.534) | 0.109 | NS | 0.746 | 0.522-1.067 |
|  |  | CC | 82(0.402) | 90(0.304) | 0.024 | NS | 1.538 | 1.059-2.236 |
|  |  | A | 150(0.368) | 254(0.429) | 0.052 | NS | 0.774 | 0.597-1.002 |
|  |  | C | 258(0.632) | 338(0.571) |  |  | 1.293 | 0.998-1.674 |
| CCL17 | rs223828 | F | 161 | 298 |  |  |  |  |
|  |  | CC | 75(0.466) | 143(0.480) | 0.774 | NS | 0.945 | 0.644-1.388 |
|  |  | CT | 69(0.429) | 128(0.429) | 0.984 | NS | 0.996 | 0.676-1.467 |
|  |  | TT | 17(0.105) | 27(0.091) | 0.603 | NS | 1.185 | 0.625-2.246 |
|  |  | C | 219(0.680) | 414(0.695) | 0.65 | NS | 0.935 | 0.698-1.252 |
|  |  | T | 103(0.320) | 182(0.305) |  |  |  |  |
|  |  | M | 195 | 320 |  |  |  |  |
|  |  | CC | 75(0.385) | 151(0.472) | 0.053 | NS | 0.7 | 0.487-1.005 |
|  |  | CT | 98(0.503) | 142(0.444) | 0.194 | NS | 1.266 | 0.886-1.810 |
|  |  | TT | 22(0.112) | 27(0.084) | 0.286 | NS | 1.38 | 0.762-2.498 |
|  |  | C | 248(0.636) | 444(0.694) | 0.055 | NS | 0.771 | 0.591-1.006 |
|  |  | T | 142(0.364) | 196(0.306) |  |  |  |  |
|  | rs223895 | F | 167 | 260 |  |  |  |  |
|  |  | CC | 48(0.287) | 76(0.292) | 0.914 | NS | 0.977 | 0.636-1.499 |
|  |  | CT | 83(0.497) | 138(0.531) | 0.496 | NS | 0.874 | 0.592-1.289 |
|  |  | TT | 36(0.216) | 46(0.177) | 0.322 | NS | 1.278 | 0.785-2.081 |
|  |  | C | 179(0.536) | 290(0.558) | 0.533 | NS | 0.916 | 0.695-1.207 |
|  |  | T | 155(0.464) | 230(0.442) |  |  | 1.092 | 0.828-1.439 |
|  |  | M | 203 | 285 |  |  |  |  |
|  |  | CC | 55(0.271) | 91(0.319) | 0.25 | NS | 0.792 | 0.533-1.179 |
|  |  | CT | 108(0.532) | 136(0.477) | 0.233 | NS | 1.246 | 0.868-1.786 |
|  |  | TT | 40(0.197) | 58(0.204) | 0.861 | NS | 0.96 | 0.612-1.507 |
|  |  | C | 218(0.537) | 318(0.558) | 0.517 | NS | 0.919 | 0.712-1.187 |
|  |  | T | 188(0.463) | 252(0.442) |  |  | 1.088 | 0.843-1.405 |
| CCL21 | rs951005 | F | 167 | 260 |  |  |  |  |
|  |  | AA | 151(0.904) | 231(0.888) | 0.605 | NS | 1.185 | 0.622-2.256 |
|  |  | AG | 16(0.096) | 28(0.108) | 0.693 | NS | 0.878 | 0.459-1.678 |
|  |  | GG | 0(0.00) | 1(0.004) | - |  | - | - |
|  |  | A | 318(0.952) | 490(0.942) | 0.536 | NS | 1.217 | 0.653-2.269 |
|  |  | G | 16(0.048) | 30(0.058) |  |  | 0.822 | 0.441-1.532 |
|  |  | M | 203 | 288 |  |  |  |  |
|  |  | AA | 189(0.931) | 256(0.889) | 0.115 | NS | 1.688 | 0.876-3.251 |
|  |  | AG | 14(0.069) | 31(0.108) | 0.144 | NS | 0.614 | 0.318-1.186 |
|  |  | GG | 0(0.00) | 1(0.003) | - |  | - | - |
|  |  | A | 392(0.966) | 543(0.943) | 0.099 | NS | 1.702 | 0.899-3.222 |
|  |  | G | 14(0.034) | 33(0.057) |  |  | 0.588 | 0.310-1.113 |
|  | rs2812378 | F | 166 | 261 |  |  |  |  |
|  |  | AA | 145(0.873) | 232(0.892) | 0.553 | NS | 0.833 | 0.456-1.522 |
|  |  | AG | 19(0.114) | 24(0.092) | 0.459 | NS | 1.271 | 0.673-2.401 |
|  |  | GG | 2(0.012) | 5(0.015) | 1 | NS | 1.281 | 0.232-7.075 |
|  |  | A | 309(0.931) | 488(0.938) | 0.654 | NS | 0.881 | 0.506-1.534 |
|  |  | G | 23(0.069) | 32(0.062) |  |  | 1.135 | 0.652-1.976 |
|  |  | M | 203 | 295 |  |  |  |  |
|  |  | AA | 187(0.921) | 262(0.888) | 0.224 | NS | 1.472 | 0.787-2.753 |
|  |  | AG | 15(0.074) | 33(0.112) | 0.158 | NS | 0.633 | 0.335-1.199 |
|  |  | GG | 1(0.005) | 0(0.00) | - |  | - | - |
|  |  | A | 389(0.958) | 557(0.994) | 0.318 | NS | 1.356 | 0.745-2.468 |
|  |  | G | 17(0.042) | 33(0.056) |  |  | 0.738 | 0.405-1.343 |
|  | rs2492358 | F | 164 | 261 |  |  |  |  |
|  |  | CC | 0(0.00) | 2(0.008) | - |  | - | - |
|  |  | CT | 15(0.091) | 28(0.107) | 0.599 | NS | 0.838 | 0.433-1.621 |
|  |  | TT | 149(0.909) | 231(0.885) | 0.444 | NS | 1.29 | 0.671-2.479 |
|  |  | C | 15(0.046) | 32(0.061) | 0.334 | NS | 0.734 | 0.391-1.377 |
|  |  | T | 313(0.954) | 490(0.939) |  |  | 1.363 | 0.726-2.557 |
|  |  | M | 204 | 295 |  |  |  |  |
|  |  | CC | 0(0.000) | 2(0.007) | - |  | - | - |
|  |  | CT | 14(0.069) | 29(0.098) | 0.245 | NS | 0.676 | 0.348-1.314 |
|  |  | TT | 190(0.931) | 264(0.895) | 0.162 | NS | 1.594 | 0.825-3.077 |
|  |  | C | 14(0.034) | 33(0.056) | 0.113 | NS | 0.6 | 0.317-1.136 |
|  |  | T | 394(0.966) | 557(0.944) |  |  | 1.667 | 0.881-3.157 |
| CCL22 | rs4359426 | F | 163 | 260 |  |  |  |  |
|  |  | AA | 2(0.012) | 4(0.015) | 1 | NS | 0.795 | 0.144-4.390 |
|  |  | AC | 47(0.288) | 69(0.265) | 0.606 | NS | 1.122 | 0.725-1.736 |
|  |  | CC | 114(0.699) | 187(0.719) | 0.661 | NS | 0.908 | 0.591-1.397 |
|  |  | A | 51(0.156) | 77(0.148) | 0.741 | NS | 1.067 | 0.726-1.567 |
|  |  | C | 275(0.844) | 443(0.852) |  |  | 0.937 | 0.638-1.377 |
|  |  | M | 199 | 286 |  |  |  |  |
|  |  | AA | 2(0.01) | 3(0.01) | 1 | NS | 0.958 | 0.159-5.785 |
|  |  | AC | 59(0.296) | 65(0.227) | 0.086 | NS | 1.433 | 0.950-2.162 |
|  |  | CC | 138(0.693) | 218(0.762) | 0.092 | NS | 0.706 | 0.470-1.059 |
|  |  | A | 63(0.158) | 71(0.124) | 0.129 | NS | 1.327 | 0.920-1.914 |
|  |  | C | 335(0.842) | 501(0.876) |  |  | 0.754 | 0.522-1.087 |
| CCL24 | rs2302004 | F | 163 | 260 |  |  |  |  |
|  |  | AA | 18(0.110) | 24(0.092) | 0.544 | NS | 1.221 | 0.640-2.327 |
|  |  | AG | 69(0.423) | 118(0.454) | 0.538 | NS | 0.883 | 0.595-1.311 |
|  |  | GG | 76(0.466) | 118(0.454) | 0.803 | NS | 1.051 | 0.710-1.557 |
|  |  | A | 105(0.322) | 166(0.319) | 0.931 | NS | 1.013 | 0.753-1.363 |
|  |  | G | 221(0.678) | 354(0.681) |  |  | 0.987 | 0.734-1.328 |
|  |  | M | 200 | 287 |  |  |  |  |
|  |  | AA | 19(0.095) | 25(0.087) | 0.765 | NS | 1.1 | 0.588-2.057 |
|  |  | AG | 78(0.390) | 115(0.401) | 0.812 | NS | 0.956 | 0.661-1.384 |
|  |  | GG | 103(0.515) | 147(0.512) | 0.951 | NS | 1.011 | 0.705-1.451 |
|  |  | A | 116(0.290) | 165(0.287) | 0.931 | NS | 1.012 | 0.764-1.342 |
|  |  | G | 284(0.710) | 409(0.713) |  |  | 0.988 | 0.745-1.309 |
|  | rs2302005 | F | 167 | 262 |  |  |  |  |
|  |  | AA | 66(0.398) | 110(0.423) | 0.602 | NS | 0.9 | 0.605-1.338 |
|  |  | AG | 82(0.494) | 120(0.462) | 0.513 | NS | 1.139 | 0.771-1.682 |
|  |  | GG | 18(0.108) | 30(0.115) | 0.825 | NS | 0.932 | 0.502-1.733 |
|  |  | A | 214(0.645) | 340(0.654) | 0.782 | NS | 0.96 | 0.720-1.281 |
|  |  | G | 118(0.355) | 180(0.346) |  |  | 1.042 | 0.781-1.390 |
|  |  | M | 204 | 296 |  |  |  |  |
|  |  | AA | 90(0.443) | 135(0.458) | 0.753 | NS | 0.944 | 0.659-1.352 |
|  |  | AG | 90(0.443) | 124(0.420) | 0.61 | NS | 1.098 | 0.766-1.575 |
|  |  | GG | 23(0.113) | 36(0.122) | 0.767 | NS | 0.919 | 0.527-1.604 |
|  |  | A | 270(0.665) | 394(0.668) | 0.927 | NS | 0.988 | 0.755-1.291 |
|  |  | G | 136(0.335) | 196(0.332) |  |  | 1.013 | 0.775-1.324 |
| CXCL8 | rs2227306 | F | 163 | 257 |  |  |  |  |
|  |  | CC | 64(0.393) | 100(0.389) | 0.942 | NS | 1.015 | 0.679-1.517 |
|  |  | CT | 70(0.429) | 122(0.475) | 0.364 | NS | 0.833 | 0.561-1.236 |
|  |  | TT | 29(0.178) | 35(0.136) | 0.246 | NS | 1.373 | 0.802-2.348 |
|  |  | C | 198(0.607) | 322(0.626) | 0.579 | NS | 0.922 | 0.693-1.227 |
|  |  | T | 128(0.393) | 192(0.374) |  |  | 1.084 | 0.815-1.442 |
|  |  | M | 203 | 294 |  |  |  |  |
|  |  | CC | 87(0.429) | 113(0.384) | 0.323 | NS | 1.201 | 0.835-1.729 |
|  |  | CT | 88(0.433) | 149(0.507) | 0.108 | NS | 0.745 | 0.520-1.067 |
|  |  | TT | 28(0.138) | 32(0.109) | 0.328 | NS | 1.31 | 0.762-2.253 |
|  |  | C | 262(0.645) | 375(0.638) | 0.807 | NS | 1.033 | 0.794-1.345 |
|  |  | T | 144(0.355) | 213(0.362) |  |  | 0.968 | 0.743-1.260 |
|  | rs2227543 | F | 163 | 259 |  |  |  |  |
|  |  | CC | 63(0.387) | 91(0.351) | 0.465 | NS | 1.163 | 0.775-1.745 |
|  |  | CT | 70(0.429) | 123(0.475) | 0.361 | NS | 0.832 | 0.561-1.235 |
|  |  | TT | 30(0.184) | 45(0.174) | 0.787 | NS | 1.073 | 0.644-1.787 |
|  |  | C | 196(0.601) | 305(0.589) | 0.72 | NS | 1.053 | 0.794-1.397 |
|  |  | T | 130(0.399) | 213(0.411) |  |  | 0.95 | 0.716-1.260 |
|  |  | M | 201 | 293 |  |  |  |  |
|  |  | CC | 83(0.413) | 96(0.328) | 0.053 | NS | 1.443 | 0.995-2.094 |
|  |  | CT | 85(0.423) | 157(0.536) | 0.014 | NS | 0.635 | 0.442-0.912 |
|  |  | TT | 33(0.164) | 40(0.137) | 0.395 | NS | 1.242 | 0.753-2.049 |
|  |  | C | 251(0.624) | 349(0.596) | 0.362 | NS | 1.129 | 0.870-1.465 |
|  |  | T | 151(0.376) | 237(0.404) |  |  | 0.886 | 0.683-1.150 |
|  | rs4694178 | F | 165 | 256 |  |  |  |  |
|  |  | AA | 57(0.345) | 84(0.328) | 0.713 | NS | 1.081 | 0.715-1.634 |
|  |  | AC | 72(0.436) | 122(0.477) | 0.419 | NS | 0.85 | 0.574-1.260 |
|  |  | CC | 36(0.218) | 50(0.195) | 0.57 | NS | 1.15 | 0.710-1.861 |
|  |  | A | 186(0.564) | 290(0.566) | 0.937 | NS | 0.989 | 0.748-1.307 |
|  |  | C | 144(0.436) | 222(0.434) |  |  | 1.011 | 0.765-1.337 |
|  |  | M | 200 | 292 |  |  |  |  |
|  |  | AA | 72(0.360) | 90(0.319) | 0.35 | NS | 1.2 | 0.819-1.759 |
|  |  | AC | 92(0.460) | 153(0.543) | 0.074 | NS | 0.718 | 0.499-1.033 |
|  |  | CC | 36(0.180) | 39(0.138) | 0.213 | NS | 1.368 | 0.834-2.242 |
|  |  | A | 236(0.590) | 333(0.590) | 0.989 | NS | 0.998 | 0.769-1.295 |
|  |  | C | 164(0.410) | 231(0.410) |  |  | 1.002 | 0.772-1.3 |
| CXCL9 | rs2276886 | F | 166 | 260 |  |  |  |  |
|  |  | CC | 62(0.373) | 86(0.331) | 0.366 | NS | 0.206 | 0.803-1.812 |
|  |  | CT | 82(0.494) | 126(0.485) | 0.85 | NS | 1.038 | 0.703-1.533 |
|  |  | TT | 22(0.133) | 48(0.185) | 0.157 | NS | 0.675 | 0.390-1.166 |
|  |  | C | 206(0.620) | 298(0.573) | 0.17 | NS | 1.218 | 0.919-1.614 |
|  |  | T | 126(0.380) | 222(0.427) |  |  | 0.821 | 0.620-1.088 |
|  |  | M | 203 | 295 |  |  |  |  |
|  |  | CC | 73(0.360) | 111(0.376) | 0.705 | NS | 0.931 | 0.642-1.349 |
|  |  | CT | 85(0.419) | 132(0.447) | 0.525 | NS | 0.89 | 0.620-1.276 |
|  |  | TT | 45(0.222) | 52(0.176) | 0.209 | NS | 1.331 | 0.852-2.080 |
|  |  | C | 231(0.569) | 354(0.600) | 0.238 | NS | 0.88 | 0.681-1.137 |
|  |  | T | 175(0.431) | 236(0.400) |  |  | 1.136 | 0.879-1.468 |
|  | rs2869460 | F | 162 | 257 |  |  |  |  |
|  |  | CC | 39(0.241) | 81(0.315) | 0.101 | NS | 0.689 | 0.441-1.076 |
|  |  | CT | 78(0.481) | 126(0.490) | 0.861 | NS | 0.965 | 0.651-1.431 |
|  |  | TT | 45(0.278) | 50(0.195) | 0.048 | NS | 1.592 | 1.003-2.528 |
|  |  | C | 156(0.481) | 288(0.560) | 0.026 | NS | 0.729 | 0.551-0.963 |
|  |  | T | 168(0.519) | 226(0.440) |  |  | 1.372 | 1.038-1.814 |
|  |  | M | 195 | 289 |  |  |  |  |
|  |  | CC | 65(0.333) | 88(0.304) | 0.503 | NS | 1.142 | 0.774-1.685 |
|  |  | CT | 82(0.421) | 123(0.426) | 0.911 | NS | 0.979 | 0.678-1.415 |
|  |  | TT | 48(0.246) | 78(0.270) | 0.559 | NS | 0.883 | 0.582-1.340 |
|  |  | C | 212(0.544) | 299(0.517) | 0.422 | NS | 1.111 | 0.859-1.438 |
|  |  | T | 178(0.456) | 279(0.483) |  |  | 0.9 | 0.696-1.164 |
| CXCL10 | rs2869462 | F | 165 | 255 |  |  |  |  |
|  |  | CC | 62(0.376) | 75(0.294) | 0.081 | NS | 1.445 | 0.954-2.187 |
|  |  | CG | 81(0.491) | 125(0.490) | 0.989 | NS | 1.003 | 0.678-1.484 |
|  |  | GG | 22(0.133) | 55(0.216) | 0.033 | NS | 0.559 | 0.326-0.959 |
|  |  | C | 205(0.621) | 275(0.539) | 0.019 | NS | 1.401 | 1.057-1.859 |
|  |  | G | 125(0.379) | 235(0.461) |  |  | 0.714 | 0.558-0.946 |
|  |  | M | 203 | 280 |  |  |  |  |
|  |  | CC | 73(0.360) | 109(0.389) | 0.506 | NS | 0.881 | 0.606-1.280 |
|  |  | CG | 85(0.419) | 118(0.421) | 0.953 | NS | 0.989 | 0.686-1.426 |
|  |  | GG | 45(0.222) | 53(0.189) | 0.382 | NS | 1.22 | 0.781-1.906 |
|  |  | C | 231(0.569) | 336(0.6) | 0.334 | NS | 0.88 | 0.679-1.140 |
|  |  | G | 175(0.431) | 224(0.4) |  |  | 1.136 | 0.877-1.473 |
| CXCL12 | rs1801157 | F | 166 | 251 |  |  |  |  |
|  |  | CC | 103(0.620) | 120(0.478) | 0.004 | NS | 1.785 | 1.197-2.661 |
|  |  | CT | 52(0.313) | 116(0.462) | 0.002 | NS | 0.531 | 0.352-0.801 |
|  |  | TT | 11(0.066) | 15(0.060) | 0.313 | NS | 1.761 | 0.579-5.360 |
|  |  | C | 258(0.777) | 356(0.709) | 0.029 | NS | 1.43 | 1.036-1.974 |
|  |  | T | 74(0.223) | 146(0.291) |  |  | 0.699 | 0.507-0.965 |
|  |  | M | 202 | 296 |  |  |  |  |
|  |  | CC | 120(0.594) | 151(0.510) | 0.065 | NS | 1.405 | 0.979-2.018 |
|  |  | CT | 70(0.347) | 125(0.422) | 0.089 | NS | 0.725 | 0.501-1.051 |
|  |  | TT | 12(0.059) | 20(0.068) | 0.715 | NS | 0.872 | 0.416-1.825 |
|  |  | C | 310(0.767) | 427(0.721) | 0.104 | NS | 1.274 | 0.951-1.707 |
|  |  | T | 94(0.233) | 165(0.279) |  |  | 0.785 | 0.586-1.051 |
|  | rs2839693 | F | 167 | 258 |  |  |  |  |
|  |  | CC | 125(0.749) | 191(0.740) | 0.85 | NS | 1.044 | 0.668-1.632 |
|  |  | CT | 42(0.251) | 66(0.256) | 0.92 | NS | 0.977 | 0.625-1.529 |
|  |  | TT | 0(0.000) | 1(0.004) | - |  | - | - |
|  |  | C | 292(0.874) | 448(0.868) | 0.798 | NS | 1.055 | 0.699-1.593 |
|  |  | T | 42(0.126) | 68(0.132) |  |  | 0.948 | 0.628-1.431 |
|  |  | M | 194 | 285 |  |  |  |  |
|  |  | CC | 143(0.737) | 207(0.726) | 0.794 | NS | 1.057 | 0.7-1.596 |
|  |  | CT | 49(0.253) | 74(0.260) | 0.862 | NS | 0.964 | 0.634-1.464 |
|  |  | TT | 2(0.010) | 4(0.014) | 1 | NS | 0.732 | 0.133-4.035 |
|  |  | C | 335(0.863) | 488(0.856) | 0.751 | NS | 1.062 | 0.732-1.541 |
|  |  | T | 53(0.137) | 82(0.144) |  |  | 0.942 | 0.649-1.366 |
| CXCL16 | rs2277680 | F | 166 | 262 |  |  |  |  |
|  |  | AA | 70(0.422) | 97(0.370) | 0.288 | NS | 1.24 | 0.834-1.845 |
|  |  | AG | 77(0.464) | 133(0.508) | 0.377 | NS | 0.839 | 0.568-1.239 |
|  |  | GG | 19(0.114) | 32(0.122) | 0.811 | NS | 0.929 | 0.508-1.700 |
|  |  | A | 217(0.654) | 327(0.624) | 0.381 | NS | 1.137 | 0.853-1.515 |
|  |  | G | 115(0.346) | 197(0.376) |  |  | 0.88 | 0.660-1.172 |
|  |  | M | 203 | 279 |  |  |  |  |
|  |  | AA | 84(0.414) | 103(0.368) | 0.306 | NS | 1.213 | 0.838-1.756 |
|  |  | AG | 101(0.498) | 143(0.511) | 0.128 | NS | 0.76 | 0.534-1.082 |
|  |  | GG | 18(0.089) | 34(0.121) | 0.252 | NS | 0.704 | 0.385-1.286 |
|  |  | A | 269(0.663) | 349(0.623) | 0.209 | NS | 1.187 | 0.909-1.551 |
|  |  | G | 137(0.337) | 211(0.377) |  |  | 0.842 | 0.645-1.101 |

**Supplemental Table 5. The best model gene-gene interaction results in BD by MDR**

| **Model** | **Bal.Acc.CV Testing** | **CV Consistency** | **P Value** |
| --- | --- | --- | --- |
| rs223828 | 0.5215 | 8/10 | 0.7 |
| rs223828, rs2869462 | 0.5074 | 5/10 | 0.8948 |
| rs2107538, rs223828, rs2869462 | 0.5137 | 5/10 | 0.8057 |

**Supplemental Table 6. The best model gene-gene interaction results in VKH by MDR**

| **Model** | **Bal.Acc.CV Testing** | **CV Consistency** | **P Value** |
| --- | --- | --- | --- |
| rs1801157 | 0.5352 | 9/10 | 0.5366 |
| rs1801157, rs2277680 | 0.5336 | 7/10 | 0.5558 |
| rs9355610, rs2869460, rs1801157 | 0.52 | 5/10 | 0.728 |
